# Gene–environment interactions and colorectal cancer risk: An umbrella review of systematic reviews and meta‐analyses of observational studies

**DOI:** 10.1002/ijc.32057

**Published:** 2019-01-16

**Authors:** Tian Yang, Xue Li, Zahra Montazeri, Julian Little, Susan M. Farrington, John P.A. Ioannidis, Malcolm G. Dunlop, Harry Campbell, Maria Timofeeva, Evropi Theodoratou

**Affiliations:** ^1^ Centre for Global Health Research, Usher Institute of Population Health Sciences and Informatics The University of Edinburgh Edinburgh United Kingdom; ^2^ School of Epidemiology and Public Health University of Ottawa Ottawa Ontario Canada; ^3^ Colon Cancer Genetics Group, Medical Research Council Human Genetics Unit, Medical Research Council Institute of Genetics & Molecular Medicine Western General Hospital, The University of Edinburgh Edinburgh United Kingdom; ^4^ Cancer Research UK Edinburgh Centre, Medical Research Council Institute of Genetics & Molecular Medicine Western General Hospital, The University of Edinburgh Edinburgh United Kingdom; ^5^ Stanford Prevention Research Center, Departments of Medicine, of Health Research and Policy, and of Biomedical Data Science, Stanford University School of Medicine, and Department of Statistics Stanford University School of Humanities and Sciences Stanford California USA; ^6^ Meta‐Research Innovation Center at Stanford (METRICS) Stanford University Stanford California USA

**Keywords:** colorectal cancer, diet, environment, gene, interaction, risk factor

## Abstract

The cause of colorectal cancer (CRC) is multifactorial, involving both genetic variants and environmental risk factors. We systematically searched the MEDLINE, EMBASE, China National Knowledge Infrastructure (CNKI) and Wanfang databases from inception to December 2016, to identify systematic reviews and meta‐analyses of observational studies that investigated gene–environment (G×E) interactions in CRC risk. Then, we critically evaluated the cumulative evidence for the G×E interactions using an extension of the Human Genome Epidemiology Network's Venice criteria. Overall, 15 articles reporting systematic reviews of observational studies on 89 G×E interactions, 20 articles reporting meta‐analyses of candidate gene‐ or single‐nucleotide polymorphism‐based studies on 521 G×E interactions, and 8 articles reporting 33 genome‐wide G×E interaction analyses were identified. On the basis of prior and observed scores, only the interaction between rs6983267 (8q24) and aspirin use was found to have a moderate overall credibility score as well as main genetic and environmental effects. Though 5 other interactions were also found to have moderate evidence, these interaction effects were tenuous due to the lack of main genetic effects and/or environmental effects. We did not find highly convincing evidence for any interactions, but several associations were found to have moderate strength of evidence. Our conclusions are based on application of the Venice criteria which were designed to provide a conservative assessment of G×E interactions and thus do not include an evaluation of biological plausibility of an observed joint effect.

**Abbreviations**
AICR: American Institute for Cancer Research
CI: confidence interval
CNKI: China National Knowledge Infrastructure
CRC: colorectal cancer
*CTNNB1*: cadherin‐associated protein β1
CUP: continuous update project
*CYP24A1*: cytochrome P450 family 24 subfamily A member 1
G×E: gene–environment
*GATA3*: GATA binding protein 3
GECCO: Genetics and Epidemiology of Colorectal Cancer Consortium
GWAS: genome‐wide association study
*HIATL1*: hippocampus abundant transcript‐like 1
*MINK1*: misshapen like kinase 1
*NAT2*: N‐acetyltransferase 2
NSAID: nonsteroidal anti‐inflammatory drug
OR: odds ratio
*PI3K*: phosphatidylinositol‐4,5‐bisphosphonate 3‐kinase
*PIK3C2G*: phosphatidylinositol‐4‐phosphate 3‐kinase catalytic subunit type 2 gamma
*PTCHD3*: patched domain containing 3
RR: relative risk
*SHMT1*: serine hydroxymethyltransferase 1
SNP: single‐nucleotide polymorphism
SOCCS: Scottish Colorectal Cancer Study
*TCF7L2*: transcription factor 7 like 2
WCRF: World Cancer Research Fund International

## Introduction

Colorectal cancer (CRC) is the third most common cancer worldwide, with 746,000 new cases in men and 614,000 new cases in women.^1^ In some low‐to‐middle‐income countries, the incidence of CRC has been increasing partly due to changes in lifestyle and environment combined with aging populations.^2^ Thus, it is critical to understand both modifiable and non‐modifiable risk factors for CRC as this may enable more specific prevention strategies and risk assessment, especially in developing countries where CRC screening may not be feasible or affordable.^3^

CRC risk is determined by a complex interplay of both genetic variants and environmental exposures. It has been speculated that their interactions ‐ known as gene–environment (G×E) interactions should also be important determinants of CRC risk. To date, genome‐wide association study (GWAS) have shown that up to 50% of CRC heritability can be explained by common and rare variants included in popular genotyping arrays.^4^ Additional variants associated with CRC susceptibility that cannot be easily detected in GWAS by marginal effects of genetic factors may be identified by testing for interactions between single‐nucleotide polymorphisms (SNPs) and environmental risk factors.^5^, ^6^ Thus, identification of G×E interactions influencing CRC susceptibility may help to discover novel genetic and environmental risk factors for CRC, and extend our understanding of biological pathways and mechanisms of cancer etiology.

A number of systematic reviews, meta‐analyses and genome‐wide G×E interaction analyses that explored interaction effects in CRC have been published. We recently collected and evaluated the evidence across existing meta‐analyses of observational studies in dietary factors and gene‐diet interactions for the 5 most common cancers.^7^ Here, we performed an umbrella review to collect, update, and assess the evidence across existing systematic reviews, meta‐analyses and genome‐wide G×E interaction analyses that have explored the joint effects between genes and a wider range of environmental exposures in CRC. Our aim is to provide an overview on the associations between G×E interactions and CRC risk and to pinpoint which of the associations have robust evidence by evaluating the strength of the evidence using predetermined guidelines.

## Methods

### Search strategy

We systematically searched the MEDLINE, EMBASE, China National Knowledge Infrastructure (CNKI) and Wanfang databases from inception to December 2016. The search strategy and Medical Subject Headings terms are displayed in Supporting Information Table S1. All identified publications went through a 2‐step review before being included. Titles were reviewed by 1 investigator (TY). Abstracts and full texts were reviewed by 2 investigators (TY and MT). Any discrepancies were resolved by discussion.

### Eligibility criteria

Three types of studies were eligible for this umbrella review: (i) systematic reviews of observational studies assessing interaction effects between genes and environmental exposures in CRC; (ii) meta‐analyses of candidate gene‐ or SNP‐based studies and analyses combining individual level data from multiple studies exploring G×E interactions and (iii) genome‐wide investigation of G×E interactions on CRC risk within GWAS consortia. We excluded reviews without explicit systematic literature searches; and systematic reviews or meta‐analyses of observational studies that explored associations between CRC risk and genes or environmental exposures only.

### Data extraction

One investigator (TY) extracted data which were then checked by a second investigator (ZM). For each eligible article, we extracted the first author, year of publication, the dietary and genetic risk factors examined, study design and the number of studies included. For meta‐analyses, we extracted the summary study‐specific relative risk estimates [relative risk (RR), odds ratio (OR)] along with the corresponding 95% confidence intervals (CIs), the number of cases and total participants, the *p* value for interaction and the *p* value (or *I* ^*2*^) for heterogeneity.

### Statistical analysis

For systematic reviews, we performed descriptive analyses and presented the authors’ main conclusions. The evaluation process is described in detail in Supporting Information methods.

For meta‐analyses of G×E interactions with a 2‐sided *p* value for interaction <0.05, or for G×E interactions reaching genome‐wide significance threshold, we used an extension of the Human Genome Epidemiology Network's Venice criteria^8^, ^9^ to evaluate the strength of the evidence (Table 1 10, 11, 12, 13, 14, 15, 16, 17). These guidelines have been used previously to assess cumulative evidence on joint effects of genes and environments on cancer risk.^7^, ^18^

**Table 1:** Description of the extension of the Human Genome Epidemiology Network's Venice criteria that were used to assess cumulative evidence on joint effects of genes and environments on cancer risk

Steps	Description
Step 1 **Score** for the strength of the **observed evidence** for the G×E interactions	First, we scored the strength of the observed evidence for the interaction between environmental exposures and genetic variants. Each G×E association was graded based on (i) the *amount* of evidence, (ii) the extent of *replication* and (iii) the *protection from bias*.
	(i) For the *amount* of evidence, the grade A, B or C was assigned when the total number of individuals in the smallest comparison group (assuming 1:1 ratio of cases and controls) in the meta‐analysis was greater than 1,000, 100–1,000, or less than 100, respectively.
	(ii) The *replication* consistency was assessed by the reported heterogeneity: grade A, *I* ^*2*^ < 25%; grade B, 25% ≤ *I* ^*2*^ ≤ 50%; grade C, *I* ^*2*^ > 50% or *p* value for heterogeneity <0.10.
	(iii) For *protection from bias* 3 aspects of G×E association were taken into account as suggested by Boffetta P *et al*.^8^: protection from bias for the environmental exposure, for the genetic analysis and for the overall interaction. Grade A means that bias, if present, may change the magnitude but not the presence of an association; grade B means that there is no evidence of bias that would invalidate an association, but important information is missing; and grade C means that there is a strong possibility of bias that would render the finding of an association invalid.
	On the basis of the combination of these 3 criteria (amount of evidence, degree of replication and protection from bias, each of which can be scored A, B and C), the epidemiological evidence for the association between G×E interaction and CRC risk was classified as strong, moderate or weak^8^ (Supporting Information Fig. S1).
Step 2 **Prior score (expected)** for G×E interactions	Second, we established a prior score category (expected) for the G×E interactions using a framework presented in Boffetta P *et al*.^*8*^, which is based on prior scores for (i) the evidence of the main environmental and (ii) the evidence of the main genetic effects (Supporting Information Table 2).
	(i) *Environmental main effect score*: We scored the main environmental effects based on the meta‐analyses of the associations between environmental factors and CRC risk that were presented in the World Cancer Research Fund International (WCRF)/American Institute for Cancer Research (AICR) Third Expert Report,^10^ the subsequent Continuous Update Project (CUP) CRC reports^11^ and the CUP CRC Systematic Literature Review 2016.^12^ For the information of environmental risk factors that was not available in the above mentioned sections, we performed an additional literature search in MEDLINE and abstracted the relevant data as summarized and presented in Supporting Information methods. We then categorized the environmental factors in terms of strength of evidence by applying previously described set of criteria.^13^ The evidence was classified as convincing (Class I), highly suggestive (Class II), suggestive (Class III) or weak evidence (Class IV) based on sample size, highly significant summary associations, the 95% prediction intervals, presence of the small‐study effect and the excess significance bias.
	(ii) *Genetic main effect score*: For the genetic main effects, a search in the National Human Genome Research Institute‐European Bioinformatics Institute catalog of GWAS,^14^ the GWAS central database^15^ and MEDLINE was conducted as described in Supporting Information methods. Subsequently, we scored the genetic associations using the Human Genome Epidemiology Network Venice criteria.^9^, ^16^, ^17^ Only genetic effects with *p* <10^−5^ were considered for evaluation, and the evidence was classified as strong, moderate or weak based on a combination of the 3 criteria (amount of evidence, degree of replication and protection from bias), each of which was scored A, B or C (Supporting Information Fig. S1). For the genetic variants that reached genome‐wide significance threshold, the evidence class of the genetic variant was only based on the amount of evidence.^16^ The search strategies, the Medical Subject Headings terms and the numbers of hits are presented in Supporting Information Tables 3 and 4, respectively.
Step 3 **Combined score**	Lastly, we examined the overall plausibility of each interaction by combining the prior score and the strength of the observed evidence. Higher weight was given to the observed evidence in case of conflicting results between the observed evidence and the prior scores.

First, we scored the strength of the observed evidence for the interaction between environmental exposures and genetic variants (observed score). Each G×E association was graded based on the *amount* of evidence, the extent of *replication* and the protection from *bias* (Table 1). On the basis of the combination of these 3 criteria, the epidemiological evidence for the association between G×E interaction and CRC risk was classified as strong, moderate or weak^8^ (Supporting Information Fig. S1).

Second, we established a prior score category (expected) for the G×E interactions using a framework presented in Boffetta *et al*.,^8^ which is based on prior scores for (i) the evidence of the main environmental and (ii) the evidence of the main genetic effects (Table 1). In brief, we established the prior score for the interactions based on the strength of evidence for the main environmental effect and the main genetic effect (1 = strong, 2 = moderate, 3 = weak) (Supporting Information Table S2). When both of the evidence were convincing (Class I), then the prior score category was strong. When one of the evidence was suggestive (Class III) or weak (Class IV), then the prior score category was weak.

Third, we examined the overall plausibility of each interaction by combining the prior score and the strength of the observed evidence. Higher weight was given to the observed evidence in case of conflicting results between the observed evidence and the prior scores.

Finally, for the statistically significant G×E interactions (with a 2‐sided *p* value for interaction <0.05) that were identified from the candidate meta‐analyses or for interactions that were concluded as suggestive by the authors of the systematic reviews, we tested the interactions in the Scottish Colorectal Cancer Study (SOCCS)^19^ dataset, and we also compared to results from the Genetics and Epidemiology of Colorectal Cancer Consortium (GECCO).^20^

## Results

### Number and type of articles identified

**Figure 1:**
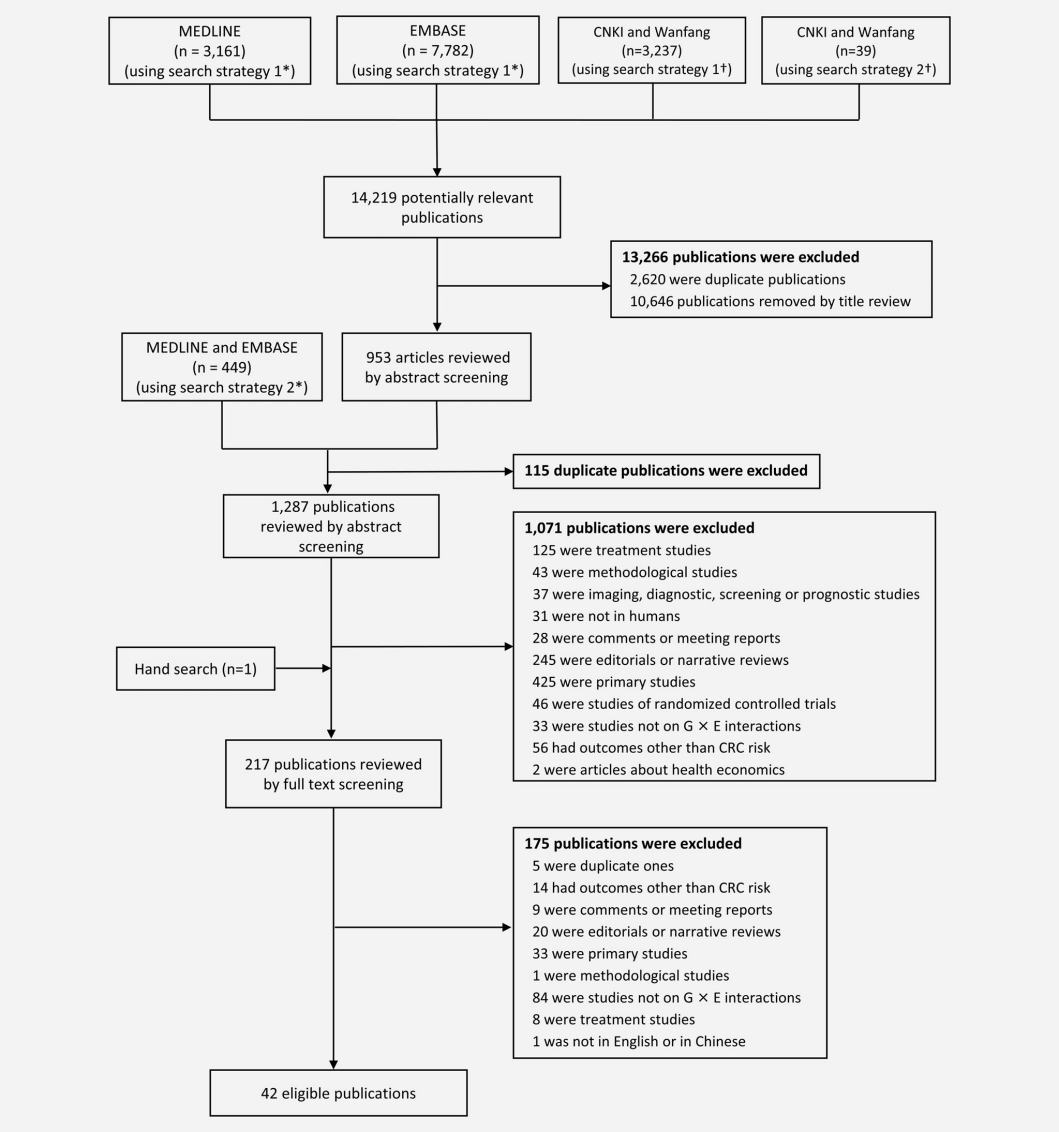
Flow chart of the literature search in MEDLINE, EMBASE, CNKI and Wanfang. *For the search in MEDLINE and EMBASE, we used both AND and OR to combine the keywords “G×E interactions” and “((gene* OR genom*) AND specific environmental risk factors)”, considering that there might be some publications that did not include the keyword “G×E interactions”. †For the search in CNKI and Wanfang, both strategies that included and not included specific environmental risk factors were used due to the limit of length of search strategies in these two databases.

Overall, 14,219 publications were identified across the 4 databases. After applying the inclusion and exclusion criteria, 42 publications were selected for inclusion (1 was in Chinese; Fig. 1). The details of 89 G×E interactions covering 22 environmental exposures identified in 15 systematic reviews of observational studies^21^, ^22^, ^23^, ^24^, ^25^, ^26^, ^27^, ^28^, ^29^, ^30^, ^31^, ^32^, ^33^, ^34^, ^35^ are presented in Supporting Information Results section.

### Main findings of meta‐analyses of candidate gene‐ or SNP‐based studies

Twenty articles^21^, ^36^, ^37^, ^38^, ^39^, ^40^, ^41^, ^42^, ^43^, ^44^, ^45^, ^46^, ^47^, ^48^, ^49^, ^50^, ^51^, ^52^, ^53^, ^54^ reporting meta‐analyses of candidate gene‐ or SNP‐based studies and analyses combining individual level data from multiple studies explored G×E interactions on CRC risk, covering 20 environmental exposures and 43 genes or genetic variants (Supporting Information Table S5). We identified 5 G×E interactions with 2‐sided *p* value for interaction <0.05 (or adjusted *p* <0.05 after accounting for multiple comparisons): *N*‐acetyltransferase 2 (*NAT2)* and processed meat intake^51^; *NAT2* and red meat intake^51^; rs16892766 (8q23.3) and vegetable consumption^39^; serine hydroxymethyltransferase 1 (*SHMT1*) C1420T polymorphism and folate intake^46^; and rs6983267 (8q24) and aspirin use^44^ (Supporting Information Table S5). Also, the interactions between the above 5 environmental exposures (processed meat, red meat, vegetables, folate, aspirin use) and approximately 2.7 million genetic variants for CRC risk were also explored in GWAS consortia^55^, ^56^, ^57^ (Supporting Information Tables S6 and S7). However, none of the interactions observed in the candidate‐based studies were detected at the genome‐wide significance level in the GWAS consortia. We also tested interactions between rs16892766 and vegetable consumption, *SHMT1* C1420T polymorphism and folate intake and rs6983267 and aspirin use in the SOCCS,^19^ and compared to the results from the GECCO.^20^ None of the associations were nominally significant (*α* = 0.05) in our data (data not presented) or in the GECCO (data not presented). Each identified G×E interaction is described in detail in Supporting Information results.

### Main findings of genome‐wide investigation of G×E interactions within GWAS consortia

Eight articles^55^, ^56^, ^57^, ^58^, ^59^, ^60^, ^61^, ^62^ corresponding to 33 genome‐wide G×E interaction analyses explored joint effects between a large number of common polymorphisms and 22 selected environmental exposures on CRC risk by using agnostic searches (a summary of all the analyses and details are presented in Supporting Information Tables S6; SNPs with the smallest *p* value for interaction from each genome‐wide G×E interaction analysis are presented in Supporting Information Table S7). The following G×E interactions were identified that reached genome‐wide significance threshold specified by the authors of the original publication: rs4143094 (10p14) and processed meat intake^55^; rs9409565 (9q22.32) and light‐to‐moderate alcohol drinking (1–28 g/day)^60^; rs2965667 (12p12.3), rs16973225 (15q25.2) and aspirin and/or nonsteroidal anti‐inflammatory drug (NSAID) use^57^; patched domain containing 3 *(PTCHD3)* at 10p12.1, misshapen like kinase 1 (*MINK1)* at 17p13.2 and NSAID use^61^; rs964293 (20q13.2) and use of estrogen plus progestogen therapy^59^; and rs1944511 (11q23.3) and overweight^62^ (Supporting Information Table S7). Each identified genome‐wide G×E interaction is described in detail in Supporting Information results.

### Evaluation of the evidence for G×E interactions with main genetic effects of a *p* < 10^−5^

**Figure 2:**
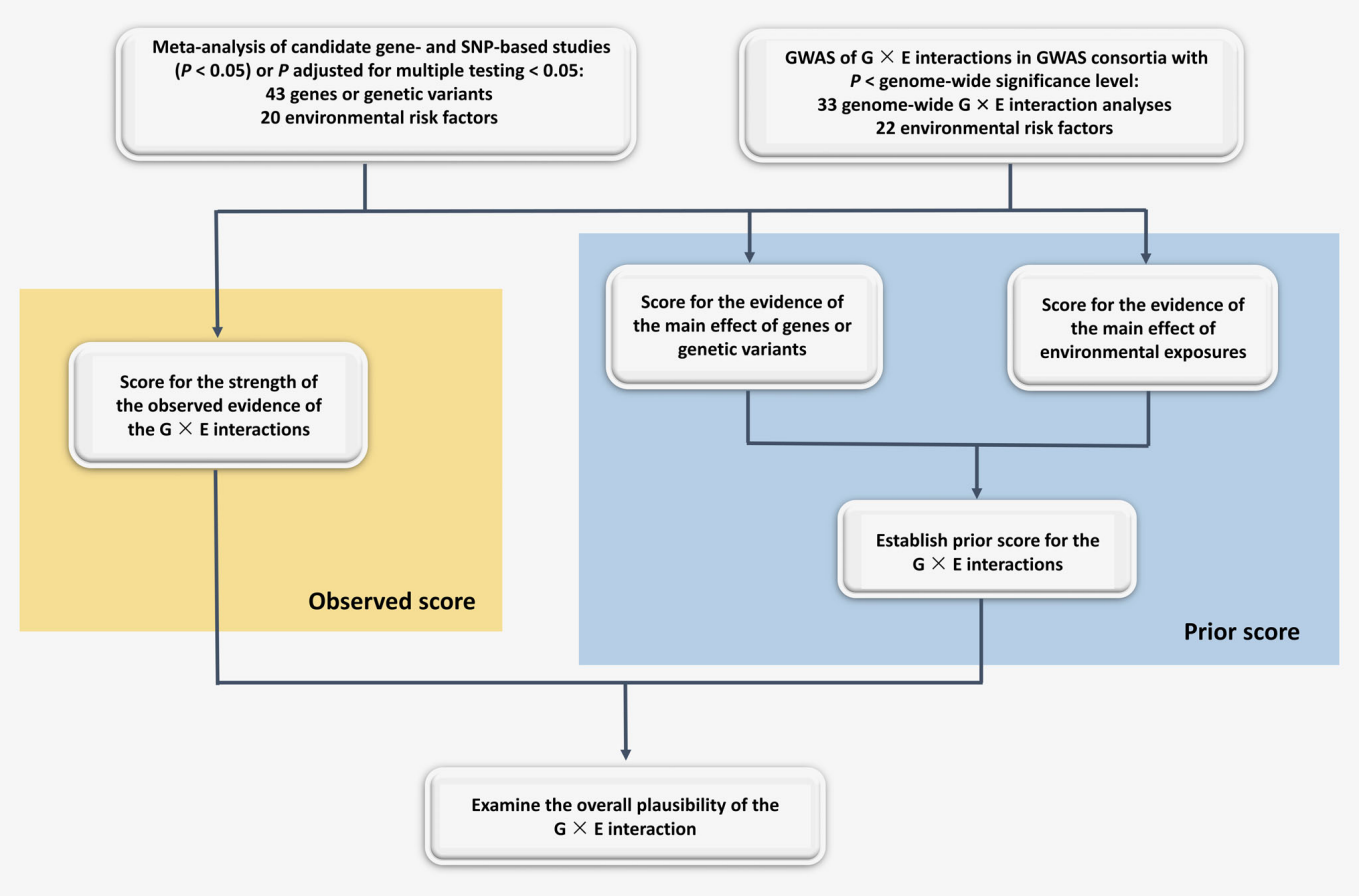
Steps in assessing G×E interactions with P for interaction < 0.05 or reached genome‐wide significance thresholds. [Color figure can be viewed at wileyonlinelibrary.com]

Here, we present the evidence for the identified G×E interactions in relation to CRC risk with main genetic effects (*p* < 10^−5^; Fig. 2).

#### *Gene‐aspirin use interactions*

Aspirin use was associated with a reduced risk of CRC on the basis of a meta‐analysis of 39 studies with 151,367 cases [users *versus* non‐users RR,0.79 (95% CI: 0.74, 0.85); *p* = 7.8×10^−11^; *I* ^*2*^ = 91.1%],^63^ thus the association was graded as highly suggestive (class II) due to the high heterogeneity between the studies (Table 2). The main effect of rs6983267 (8q24) on CRC risk was graded as strong (ABA, equivalent to AAA based on the Venice criteria^16^) in a meta‐analysis including 13,348 cases and 26,438 controls of European ancestry [OR, 0.84 (95% CI: 0.80, 0.88); *p* = 7.45×10^−13^; *I* ^*2*^ = 37.7%]^64^ (Table 3). Consequently, the interaction between rs6983267 (8q24) and aspirin use was given a moderate prior score (Moderate – 2) and a moderate overall plausibility score (Table 4).

**Table 2:** Evaluation of environmental main effects for the environmental exposures identified in the selected G×E interactions

Environmental exposure	Reference	Unit of comparison	Number of cases	Number of studies	Relative risk (95% CI)	*p* Value	Prediction interval	Heterogeneity (*I* ^*2*^ and/or *p* value)	Evidence class^1^
Processed meat	WCRF, CUP 2017	Per 50 g/day	10,738	10	1.16 (1.08, 1.26)	0.0002	NA	*I* ^*2*^ *= 20.1%;* *p* = *0.258*	III
Red meat	WCRF, CUP 2017	Per 100 g/day	6,662	8	1.12 (1.00, 1.25)	0.05	NA	*I* ^*2*^ *= 23.6%;* *p* = *0.241*	IV
Light‐to‐moderate alcohol intake	WCRF, CUP 2017	1 drink/day	36,942	8	1.06 (1.00, 1.11)	0.03	NA	*I* ^*2*^ *= 60.4%;* *p* = *0.013*	IV
Vegetables	WCRF, CUP 2017	100 g/day	14,136	11	0.98 (0.96, 0.99)	0.01	NA	*I* ^*2*^ *= 0.0%;* *p* = *0.48*	IV
Total folate	WCRF, CUP 2017	100 mcg/day	4,633	8	0.99 (0.98, 1.00)	0.05	NA	*I* ^*2*^ *= 0.0%;* *p* = *0.92*	IV
Aspirin use	Qiao Y, 2018	Users *vs*. non‐users	151,367	39	0.79 (0.74, 0.85)	7.8×10^−11^	NA	*I* ^*2*^ *=* 91.1%; *p* = 0.000	II^2^
Aspirin and/or NSAID use	Nan H, 2015	Regular users of aspirin, NSAIDs or both *vs*. non‐regular users	8,634	10	0.69 (0.64, 0.74)	6.20×10^−28^	NA	*p* = 0.02	II^2^
Use of estrogen plus progestogen therapy	Lin KJ, 2012	Ever users *vs*. non‐users	NR	17	0.74 (0.68, 0.81)	<0.001	NA	*I* ^*2*^ *= 0%;* *p* = *0.88*	III
Overweight	WCRF, CUP 2017	Per 5 kg/m^2^ increase in BMI	71,089 (total number)	38 (20 for men and 24 for women)	Men: 1.08 (1.04, 1.11); women: 1.05 (1.02, 1.08)	Men: *p* < 0.001 Women: *p* < 0.001	NA	*Men: I* ^*2*^ *= 83.3%*, *p* < *0.001;* *women*: *I* ^*2*^ *= 82.5%*, *p* < *0.001*	Men: III; Women: III

Abbreviations: BMI, body mass index; CI, confidence interval; CUP, Continuous Update Project; G×E, gene–environment; NA, not applicable; NR, not reported; NSAID, nonsteroidal anti‐inflammatory drug; *vs*., *versus*; WCRF, World Cancer Research Fund.

1 Evidence class was decided using the after criteria: Convincing evidence (class I) required >1,000 cases, highly significant summary associations (*p* < 10^−6^ by random effects), a 95% prediction interval not including the null, no evidence of small‐study effects, no evidence of excess significance bias, and low heterogeneity values (*I* ^*2*^ < 50%). Highly suggestive evidence (class II) required >1,000 cases, highly significant summary associations (*p* < 10^−6^ by random effects), and the largest study to have a 95% confidence interval that excluded 1. Suggestive evidence (class III) required only >1,000 cases and *p* < 0.001 by random effects. Evidence was considered weak (class IV) when *p* < 0.05. No association indicates evidence for the main environmental effects with *p* > 0.05.

2 The evidence was classified as highly suggestive (class II) due to the high heterogeneity between the studies.

**Table 3:** Evaluation of genetic evidence for variants identified in the selected G×E interactions

Genetic variant	Gene (or near gene)	Reference	Discovery sample size	Replication sample size / Number of studies in meta‐analysis	Reported OR (95% CI)	*p* Value for genetic main effect	Heterogeneity, *I* ^*2*^	Venice criteria	Evidence class^1^
rs4143094	10p14/*GATA3*	Figueiredo JC, 2014	9,287 cases and 9,117 controls of European ancestry from USA, Australia, Canada and Germany	Meta‐analysis, 10 studies	NR	0.26	NR	NA	No association
Slow/intermediate/rapid	*NAT2*	Zhang L, 2012	13,606 cases and 17,957 controls of Africans, Asians, Caucasians and mixed populations	Meta‐analysis, 39 studies	Slow *vs*. rapid phenotype: 0.96 (0.90, 1.01)	No association	*I* ^*2*^ = 17.8%	NA	No association
Slow/intermediate/rapid	*NAT2*	Wang H, 2015	2,186 cases and 3,736 controls of Japanese; 466 cases and 4,356 controls of African Americans	Meta‐analysis, 7 studies	Rapid *vs*. slow phenotype: Japanese: 1.05 (0.87, 1.27); African Americans: 0.75 (0.50, 1.14); Combined: 0.99 (0.83, 1.18)	Japanese: 0.77; African Americans: 0.19; Combined: 0.81	NR	NA	No association
rs9409565	9q22.32/*HIATL1*	Schumacher FR, 2015^2^	18,299 cases and 19,656 controls of European ancestry from North America, Australia and Europe	Meta‐analysis, 4,725 cases and 9,969 controls of East Asian ancestry from Republic of Korea, China and Japan	0.98 (0.95, 1.01)	0.127	NR	NA	No association
rs16892766	8q23.3/*EIF3H*	Li M, 2015	41,728 cases and 44,393 controls	Meta‐analysis, 11 studies	1.22 (1.18, 1.27)	1.39 × 10^−24^	*I* ^*2*^ = 4%	AAA	Strong
rs6983267	8q24.21	Tanskanen T, 2017	1,701 Finnish cases and 14,082 population‐based, cancer‐free controls	Meta‐analysis, 13,348 cases and 26,438 controls of European ancestry	0.84 (0.80, 0.88)	7.45 × 10^−13^	*I* ^*2*^ = 37.7%	ABA (equivalent to AAA)	Strong
C1420T	*SHMT1*	Wang Q, 2014	3,912 cases and 4,954 controls	Meta‐analysis, 7 studies	TT *vs*. CC: 0.84 (0.73, 0.97); CT *vs*. CC: 1.01 (0.92, 1.10); TT + CT *vs*. CC: 0.97 (0.89, 1.06); TT *vs*. CT + CC: 0.84 (0.73, 0.96)	TT *vs*. CC: 0.020; CT *vs*. CC: 0.903; TT + CT *vs*. CC: 0.476; TT *vs*. CT + CC: 0.013	TT *vs*. CC: *I* ^*2*^ = 3.8%; CT *vs*. CC: *I* ^*2*^ = 0%; TT + CT *vs*. CC: *I* ^*2*^ = 0%; TT *vs*. CT + CC: *I* ^*2*^ = 0%	NA	No association
rs2965667	12p12.3/*PIK3C2G*	Orlando G, 2016	8,749 cases and 18,245 controls from Europe	Meta‐analysis, 7 studies	0.97 (0.87, 1.08)	0.552	*I* ^*2*^ = 4.8%	NA	No association
rs16973225	15q25.2/*interleukin 16*	Orlando G, 2016	8,749 cases and 18,245 controls from Europe	Meta‐analysis, 7 studies	1.05 (0.97, 1.15)	0.242	*I* ^*2*^ = 0%	NA	No association
rs964293	20q13.2/*CYP24A1*	Orlando G, 2016	8,749 cases and 18,245 controls from Europe	Meta‐analysis, 7 studies	0.97 (0.93, 1.01)	0.156	*I* ^*2*^ = 6.3%	NA	No association
Including 7 variants^3^	10p12.1/*PTCHD3*	Timofeeva M, 2015	8,100 cases and 21,820 controls from Europe	Meta‐analysis, 6 studies	NR	0.352	NR	NA	No association
Including 8 variants^4^	17p13.2/*MINK1*	Timofeeva M, 2015	8,100 cases and 21,820 controls from Europe	Meta‐analysis, 6 studies	NR	0.381	NR	NA	No association
rs1944511	11q23.3	Siegert S, 2013	259 cases and 1,002 controls	Genome‐wide G×E interaction analysis	1.07	0.536	NR	NA	No association

Abbreviations: CI, confidence interval; G×E, gene–environment; NA, not applicable; NR, not reported; OR, odds ratio; *vs*., *versus*.

1 Evidence class was decided on the basis of the Human Genome Epidemiology Network's Venice criteria: No association indicates evidence for main genetic effects with *p* > 10^−5^. Only genetic effects with *p* < 10^−5^ were considered for evaluation. On the basis of a combination of 3 criteria (amount of evidence, degree of replication, and protection from bias) (each of which can be scored A, B, or C), the epidemiological evidence for an effect of the genotype is classified as strong, moderate, or weak. For amount of evidence, a grade of A, B, or C was assigned when the sample size for the rarer genotype in the meta‐analyses was greater than 1,000, 100–1,000, or less than 100, respectively. For replication consistency, we used *I* ^*2*^ < 25% to assign grade A, 25–50% to assign grade B, and > 50% or a *p* value for heterogeneity <0.10 to assign grade C. For protection from bias, a grade of A means that bias, if present, may change the magnitude but not the presence of an association; a grade of B means that there is no evidence of bias that would invalidate an association, but important information is missing; and a grade of C means that there is a strong possibility of bias that would render the finding of an association invalid. For the genetic variants that reached genome‐wide significance threshold, the evidence class of the genetic variant was only based on the amount of evidence based on the clarification of Venice Criteria (Khoury MJ et al, 2009).

2 Current study exploring the marginal association of rs9409565 was used since it is about twice as large as Gong *et al*. (2016).

3 Seven variants at 10p12.1 were included in the analysis that explored main genetic effects by Timofeeva M *et al*, 2015. However, the interaction analysis (by Jiao S *et al*, 2015) included 8 variants: chr10:27687284, chr10:27687437, chr10:27687638, chr10:27687775, chr10:27687989, chr10:27688101, chr10:27702174 and chr10:27702624.

4 Eight variants at 17p13.2 were included in the analysis that explored main genetic effects Timofeeva M *et al*, 2015. However, the interaction analysis (by Jiao S *et al*, 2015) included 4 variants: chr17:4794313, chr17:4794407, chr17:4796839 and chr17:4797910.

**Table 4:** Evaluation of evidence for the selected G×E interactions in relation to CRC risk

Environmental exposure	Genetic variant	Gene (or near gene)	Score based on observed evidence^1^	Strength of observed evidence for interaction	Score for environmental evidence (evidence class)	*p* Value for main genetic effect	Score for genetic evidence /Venice criteria^2^	Prior score^3^	Combined score^4^
*Meta‐analyses of candidate gene‐ or SNP‐based studies*									
Processed meat	Slow/intermediate/rapid	*NAT2*	(B or C) BC	Weak	III	No association	No association	Weak: 3	No evidence
Red meat	Slow/intermediate/rapid	*NAT2*	(B or C) AC	Weak	IV	No association	No association	Weak: 3	No evidence
Vegetables	rs16892766	8q23.3/*EIF3H*	CBB	Weak	IV	1.39 × 10^−24^	Strong/AAA	Weak: 3	No evidence
Folate intake	C1420T	*SHMT1*	CAC	Weak	IV	TT *vs*. CC: 0.020; CT *vs*. CC: 0.903; TT + CT *vs*. CC: 0.476; TT *vs*. CT + CC: 0.013	No association	Weak: 3	No evidence
Aspirin use	rs6983267	8q24	BAB^5^	Moderate	II	7.45 × 10^−13^	Strong/ABA (equivalent to AAA)	Moderate: 2	Moderate
*Genome‐wide G×E interaction analyses*									
Processed meat	rs4143094	10p14/*GATA3*	BBB	Moderate	III	0.26	No association	Weak: 3	Moderate
Light‐to‐moderate drinking	rs9409565	9q22.32/*HIATL1*	BBA	Moderate	IV	0.127	No association	Weak: 3	Moderate
Aspirin and/or NSAID use	rs2965667	12p12.3/*PIK3C2G*	B ‐ A	Moderate	II	0.552	No association	Weak: 3	Moderate
	rs16973225	15q25.2/*interleukin 16*	B ‐ A	Moderate	II	0.242	No association	Weak: 3	Moderate
NSAID use	Including 8 variants	10p12.1/*PTCHD3*	‐ ‐ B	Not possible to evaluate^6^	II	0.352	No association	Weak: 3	Not possible to evaluate
	Including 4 variants	17p13.2/*MINK1*	‐ ‐ B	Not possible to evaluate^6^	II	0.381	No association	Weak: 3	Not possible to evaluate
Use of estrogen plus progestogen therapy	rs964293	20q13.2/*CYP24A1*	BBA	Moderate	III	0.156	No association	Weak: 3	Moderate
Overweight	rs1944511	11q23.3	C ‐ B	Weak	III	0.536	No association	Weak: 3	No evidence

Abbreviation: CRC, colorectal cancer; G×E, gene–environment; NSAID, nonsteroidal anti‐inflammatory drug; SNP, single‐nucleotide polymorphisms; *vs*., *versus*.

1 The strength of the observed evidence for interaction between the environmental exposures and the genetic variants was based on an extension of the Human Genome Epidemiology Network's Venice criteria used for assessing cumulative evidence for genetic associations. Each G×E association was graded based on the amount of evidence, the extent of replication and protection of bias. Dashes indicate that 1, 2, or 3 elements of the Venice criteria cannot be decided. A complete score should have 3 letters, corresponding to amount of evidence, degree of replication, and protection from bias components of the Venice criteria. If 1 element is missing, the score is represented by a single dash and 2 letters. If 2 elements are missing, the score is represented by 2 dashes and a letter.

2 No association indicates evidence for main genetic effects with *p* > 10^−5^. Only genetic effects with *p* < 10^−5^ were considered for evaluation using the Human Genome Epidemiology Network's Venice criteria.

3 The prior score was based on scores for environmental evidence and genetic evidence (Table 2 and Table 3).

4 The overall plausibility of an interaction was examined by comparing the prior score and the score for the strength of the observed evidence. Higher weight was given to the observed evidence in case of conflicting results between the prior and observed scores.

5 The replication consistency was graded as A because the interaction between aspirin use and rs6983267 was replicated in the GECCO.

6 Jiao *et al* (2015) presented no information on the total number of individuals in the smallest comparison group and heterogeneity between the studies. Therefore evaluating the amount of evidence and the extent of replication according to the Venice criteria was not possible.

#### *Gene‐vegetable interactions*

A meta‐analysis of the association between vegetable intake and CRC risk in the latest Continuous Update Project (CUP) CRC reports^11^ and the CUP Colorectal Systematic Literature Review 2016^12^ showed a reduction in CRC risk with 100 g/day increase in vegetable intake [RR, 0.98 (95% CI: 0.96, 0.99); *p* = 0.01; *I* ^*2*^ = 0%; n = 11 prospective studies; n of cases = 14,136] and the association was classified as class IV (weak) (Table 2). On the basis of the strong (AAA, based on the Venice criteria^16^) main genetic (Table 3) and weak (class IV) environmental effects of vegetable intake on CRC risk, the possible interactions between 8q23.3 locus and vegetable intake on CRC risk was given a weak (Weak – 3) prior score and therefore no evidence was found for this interaction^7^ (Table 4).

### Evaluation of the evidence for G×E interactions with no main genetic effects (*p* > 10^−5^)

Here, we present the evidence for the identified G×E interactions in relation to CRC risk with no main genetic effects (*p* > 10^−5^; Fig. 2). The G×E interactions were considered as tenuous even if they were classified as having moderate evidence.

#### *Interactions between genetic variants and use of aspirin, NSAIDs or both*

In a meta‐analysis of 10 studies including 8,634 cases and 8,553 controls, regular use of aspirin and/or NSAIDs, compared to non‐regular use, was associated with lower risk of CRC [RR 0.69 (95% CI: 0.64, 0.74); *p* = 6.20 × 10^−28^; *p*_heterogeneity_ *= 0.02*], thus the evidence was classified as highly suggestive (class II) due to the high heterogeneity between the studies^57^ (Table 2). For the main genetic effects, no associations were observed between rs2965667 (12p12.3), rs16973225 (15q25.2) and CRC risk (*p* > 10^−5^) in a meta‐analysis of 7 GWAS from Europe including 8,749 cases and 18,245 controls (*p* = 0.552 and 0.242, respectively)^65^ (Table 3). Thus, the interactions between rs2965667 (12p12.3), rs16973225 (15q25.2) and aspirin and/or NSAID use were given moderate overall plausibility scores and weak (Weak – 3) prior scores (Table 4). Nevertheless, the overall plausibility scores for the interactions between 10p12.1/*PTCHD3*, 17p13.2*/MINK1* and NSAID use could not be properly evaluated due to the missing elements of the extension of the Venice criteria^8^ that was used for assessing the observed evidence for the interactions (Table 4).

#### *Interactions between genetic variants and use of estrogen plus progestogen therapy*

The RR for use of estrogen plus progestogen therapy on CRC risk was 0.74 (95% CI: 0.68, 0.81; *p* < 0.001; *I* ^*2*^ = 0%) in a meta‐analysis of 17 studies,^66^ thus the association was classified as suggestive (class III; Table 2). Furthermore, no association was observed between the rs964293 variant and CRC risk (*p* > 10^−5^) in the meta‐analysis of 7 GWAS [OR, 0.97 (95% CI: 0.93, 1.01); *p* = 0.156; *I* ^*2*^ = 6.3%]^65^ (Table 3). On the basis of the prior and observed scores, the interaction between rs964293 (20q13.2) and use of estrogen plus progestogen therapy was given a moderate overall plausibility score and a weak (Weak – 3) prior score (Table 4).

#### *Gene‐alcohol interactions*

A meta‐analysis of 8 prospective studies in the latest CUP CRC reports^11^ and the CUP Colorectal Systematic Literature Review 2016^12^ showed one drink per day increase was associated with increased CRC risk [RR, 1.06 (95% CI: 1.00, 1.11); *p* = 0.03; *I* ^*2*^ = 60.4%; n of cases = 36,942], and the association between light‐to‐moderate drinking and CRC risk was categorized as weak (class IV) (Table 2). Additionally, no main effect was observed for the rs9409565 (9q22.32) on CRC risk (*p* > 10^−5^) of 18,299 cases [OR, 0.98 (95% CI: 0.95, 1.01); *p* = 0.127]^67^ (Table 3). Hence, the interaction between rs9409565 (9q22.32) and light‐to‐moderate drinking was given a weak prior score (Weak – 3) and a moderate overall plausibility score (Table 4).

#### *Gene‐meat interactions*

No evidence was found for the interactions between processed meat, red meat and *NAT2* based on the weak (Weak – 3) prior score and the weak observed score (Tables 2, 3, 4). The possible 10p14 locus‐processed meat interaction was given a weak prior score (Weak – 3) and a moderate plausibility score^7^ (Tables 2, 3, 4).

#### *Gene‐folate interactions*

No evidence was found for the interaction between *SHMT1* C1420T and folate intake on CRC risk based on a weak (Weak – 3) prior score and a weak observed score (Tables 2, 3, 4).

#### *Gene‐overweight interactions*

No evidence was found for the interaction between rs1944511 (11q23.3) and overweight based on a weak (Weak – 3) prior score and a weak observed score (Tables 2, 3, 4).

## Discussion

### Main findings

Based on the prior and observed scores, only the interaction between rs6983267 (8q24) and aspirin use was found with a moderate overall plausibility score and a main genetic effect (*p* = 7.45 × 10^−13^; strong, based on the Venice criteria). In particular, the benefit of regular aspirin use on CRC risk was confined to individuals with T allele of rs6983267, which has been associated with impaired binding of cadherin‐associated protein β1 (*CTNNB1*)/ transcription factor 7 like 2 (*TCF7L2*) and lower expression of *MYC*.^68^, ^69^, ^70^ Moreover, aspirin has been associated with Wnt pathway and the inhibition of nuclear *CTNNB1* expression in colon cancer cell lines.^71^ Hence, it is suggested that a genetic background by which *CTNNB1/TCF7L2* binding is not constitutively active is necessary for the susceptibility to the effects of aspirin on the Wnt/*CTNNB1* pathway.^44^

Moderate overall plausibility scores were also found for the interactions between rs4143094 (10p14) and processed meat intake, rs9409565 (9q22.32) and light‐to‐moderate alcohol drinking (1–28 g/day), rs964293 (20q13.2) and use of estrogen plus progestogen therapy, as well as rs2965667 (12p12.3), rs16973225 (15q25.2) and aspirin and/or NSAID use. However, these interactions are regarded as tenuous due to the lack of main genetic effects (*p* > 10^−5^), even though they may provide clues to discovering novel CRC susceptibility loci that have not been readily detected in GWAS by their marginal effects of genetic factors.^5^, ^6^

Little is known about the underlying molecular mechanisms of the interactions between rs2965667 at the 12p12.3/ phosphatidylinositol‐4‐phosphate 3‐kinase catalytic subunit type 2 gamma (*PIK3C2G*) locus, rs16973225 (15q25.2/*interleukin 16*) and aspirin and/or NSAID use on CRC risk. *PIK3C2G* gene encodes a protein of the phosphatidylinositol‐4,5‐bisphosphonate 3‐kinase (*PI3K*) family,^72^ of which the activated signaling can inhibit apoptosis in colon cancer cell lines that can be restored with NSAID‐mediated blockade of *PI3K*.^57^, ^73^ *Interleukin 16* may stimulate monocyte induction of proinflammatory cytokines associated with tumorigenesis,^74^, ^75^ which suggests that polymorphisms in or near the *interleukin 16* gene may be associated with the production of inflammatory cytokines that modify the chemopreventive effect of aspirin and/or NSAIDs on CRC.^57^ However, it is proposed that those GWAS‐identified promising loci outside of a known coding region may affect more distant genes rather than the closest gene.^76^

The interaction between use of estrogen plus progestogen hormone preparations and the rs964293 variant in the 20q13.2, known as cytochrome P450 family 24 sub‐family A member 1 (*CYP24A1)* is biologically plausible. *CYP24A1* is greatly expressed in malignant colon tumours,^77^ and some variants in *CYP24A1* have been associated with CRC risk.^78^ In a recent meta‐analysis, ever use of estrogen plus progestogen therapy has been associated with lower CRC risk [OR: 0.74 (95% CI: 0.68, 0.81); *p* <0.001].^66^ Also, an inverse association was found in the meta‐analysis of randomized controlled trials with a hazard ratio of 0.77 (95% CI: 0.59, 0.98) (*p* = 0.037).^66^ It is suggested that *CYP24A1* may only be a metabolizing enzyme for progestrogens but not estrogen, since an interaction effect was only found with the use of combined estrogen‐progestogen therapy and not with estrogen‐only intake.^59^

The mechanism of the modifying effect of the rs4143094 variant at the 10p14 locus, near GATA binding protein 3 (*GATA3)* region, on the association between processed meat intake and CRC risk is even less clear. *GATA3* has been described as a master regulator of T‐helper 2 cell differentiation in mature CD4 (+) T cells and has been associated with T cell development.^79^ One possible explanation of the functional impact is that processed meat could trigger a pro‐tumorigenic inflammatory or immunological response,^55^, ^80^ and loss of *GATA* genes or silencing of expression can increase CRC risk.^81^

Furthermore, the mechanism of the modifying effect of alcohol consumption on the association between rs9409565 at the 9q22.32/ Hippocampus Abundant Transcript‐Like 1 *(HIATL1)* locus and CRC risk has not been understood. *HIATL1* is a member of the solute carrier group of membrane transport, which makes the move of substances (such as peptides, amino acids, proteins, metals, and neurotransmitters) directly into or out of cells possible.^82^, ^83^ Gene expression analyses indicate that the variants at 9q22.32/*HIATL1* that interact with alcohol on CRC risk through genome‐wide G×E interaction analyses can also impact *HIATL1* expression,^60^ which suggests that alcohol may modify the effects of *HIATL1* on CRC risk through its influence on *HIATL1* expression levels.^60^

### Challenges for G×E interaction studies

Studies of G×E interactions require much larger sample sizes than main effect analyses due to small effect sizes, multiple testing, misclassification due to imperfect measures of environmental exposures and more model parameters.^5^, ^84^, ^85^, ^86^ Even if the sample size is large enough to detect interactions with common exposures, it may still be insufficient when analyzing relatively rare exposures or genotypes of interest.^5^ Meta‐analyses of existing G×E interactions can address this sample size limitation, though investigators should be aware of other issues, such as i) inconsistencies in the definitions of exposures and outcomes and ii) differences in study designs, tools for assessing exposures, distributions of exposures, statistical analyses, presentation of results and publication bias.^87^ Additionally, the scale and distribution of environmental exposures in a population can also influence power. In dietary studies, it is suggested that the diet under investigation should be sufficiently variable in the population to allow evaluation of various intakes, and risk should be reported per similar units of exposure (e.g. per 100 g meat intake per day) to ensure that comparisons between populations is possible.^21^ In studies performed under the framework of GWAS consortia, the nature of exposure assessment may be different from other studies. Thus, harmonization of exposure may have been more difficult. Consequently, an increased sample size and decreased quality of exposure data, as well as a fully validated design may help to address the measurement error issue.^5^

### Strengths and limitations of current review

The strengths of umbrella reviews have been described elsewhere.^13^, ^88^, ^89^ In our study, we found moderate evidence for some G×E interactions on CRC risk, though most of these interaction effects were tenuous due to the lack of main genetic effects and/or environmental effects. The proposed biological mechanisms for the G×E interactions are hypothetical and in the absence of experimental studies could not be used to prove causality. Ideally, evidence from a model system or/and organism with genetic variations in the gene/polymorphisms of interest and exposed to the physiological dose of the environmental factor (aspirin, processed meat, alcohol, and sex hormone) is required to support the epidemiological evidence described here. Thus, further replication and functional studies are required to confirm our findings and understand the biologic implications of the interactions.

Our study has limitations. First, interaction effects that have not yet been assessed through meta‐analyses or systematic reviews would not have been included, since umbrella reviews do not focus on individual studies. Second, we did not use an established tool to assess the risk of bias in the included observational studies, because available tools such as Q‐Genie^90^ and the Newcastle‐Ottawa Scale do not capture aspects relevant to the G×E assessment. Third, we excluded reviews without explicit systematic literature searches in order to avoid bias, but this could have resulted in the exclusion of syntheses of literature that have not been systematic.^91^ Fourth, interactions with limited evidence or limited sample size may have led to false‐negative findings for some joint effects that have long been thought to exist. False‐positives may also exist, although we have applied our criteria to assess the evidence to minimize biases. In our study, we used *I* ^*2*^ to assess the heterogeneity in the evaluation, however, it has been reported that *I* ^*2*^ represents what proportion of the observed variance would remain if we could eliminate the sampling error rather than how much the effect size varies.^92^ Additionally, we would miss interactions in which there were no marginal effects of genotype or exposure on CRC risk, since the Venice criteria only aim to grade the credibility of evidence of significant main effects; and 2 of the identified G×E interactions in our study could not be properly evaluated due to lack of information required to apply these criteria.

We combined systematic literature reviews, candidate and genome‐wide G×E studies together. The main reason for this was to provide a comprehensive overview of the existing literature on G×E interaction in relation to CRC risk. However, an issue is that in each of these types of studies, different criteria and *p* value thresholds are typically used to evaluate the creditability of findings. Candidate gene studies with liberal *p* value thresholds might be biased toward false positive findings, while the genome‐wide approach is prone to false negative observations, because true interactions may not reach a stringent genome‐wide significance threshold. This, as well as other issues such as genotyping and imputation problems, could be a reason why none of the interactions identified in the candidate gene studies were replicated in genome‐wide G×E studies.

The extension of Venice criteria used here has been applied in the past to assess joint effects of environments and genes on risk of multiple cancers.^7^, ^10^ This method does not take into account other lines of evidence, such as biological plausibility of observed associations, biological gradient of effects, coherence of the observations across multiple type of studies or support by experiments. Although other guidelines are more comprehensive in the range of evidence considered such as the newly developed “integrative research” method that combines causal criteria of Austin Bradford Hill with graphical models,^93^ we did not use it to evaluate the evidence in our study, since some of the criteria are difficult to apply and interpret in molecular epidemiology (e.g. temporality).^94^

## Conclusions

Our assessment maps the status of evidence on the associations between G×E interactions and CRC risk. Despite the identified studies exploring a wide variety of G×E interactions on CRC, we conclude that we did not find highly convincing evidence for any interactions, but several associations were found to have moderate strength of evidence using our set of guidelines.

Though most of the evaluated G×E interactions in our study were with no main genetic effects, it has been suggested that such kind of risk loci that have not been readily detected in traditional GWAS may be identified by testing for interactions between SNPs and environmental risk factors, even though there is no strong evidence for a G×E interaction.^5^, ^6^ Thus, studies incorporating accurate assessment of environmental exposures are encouraged not only to identify novel G×E interactions, but also to discover novel risk loci for CRC by characterizing any underlying G×E interactions.^5^ Moreover, there remains insufficient evidence for G×E interactions on CRC risk, and some G×E interactions without strong evidence may still be important in CRC prevention. Hence, studies with large sample sizes and further functional studies are required to identify important G×E interactions that could have public health impact, so as to shed light to CRC etiology and to allow for more specific risk assessment for early‐detection or prevention strategies.

## Conflict of interest

None declared.

## Ethical approval

Not required.

## Data sharing

No additional data available.

## Authors’ contributions

Study design: MT and ET; Literature search: TY; Study selection: TY and MT; Data extraction: TY and ZM; Data analysis: TY and MT; Study draft and revision: TY, MT, ET, XL, ZM, JL, SMF, JPAI, MGD and HC; Article guarantor: Dr. Maria Timofeeva and Dr. Evropi Theodoratou.

## Supporting information


**Appendix S1:** Supplementary methods

**Figure S1 Categories for the credibility of cumulative epidemiological evidence.** The 3 letters correspond (in order) to amount of evidence, replication and protection from bias. Evidence is categorized as strong, when there is A for all 3 items, and is categorized as weak when there is a C for any of the 3 items. All other combinations are categorized as moderate (from Boffetta *et al* 2012).
**Table S1.** Keywords and Search Strategies for Meta‐analyses of G × E Interactions used in the Umbrella Review.
**Table S2.** Score Categories for Credibility of an Interaction Between an Environmental Exposure and a Genetic Variant Based on the Strength of Evidence for a Main Effect of Each of Them (1 = Strong, 2 = Moderate, 3 = Weak) (adapted from Boffetta *et al*. 2012).
**Table S3.** Search Strategy Used for the Identification of Main Environmental Effects for Colorectal Cancer Risk.
**Table S4.** Search Strategy Used for the Identification of Main Genetic Effects for Colorectal Cancer Risk.
**Table S5.** G × E Interactions in Relation to CRC Risk That Were Reported From Meta‐analyses of Candidate Gene‐ or SNP‐based Studies.
**Table S6.** General Characteristics of 33 Genome‐wide G × E Interaction Analyses in GWAS Consortia.
**Table S7.** SNPs With the Smallest *P* for G × E Interactions in Relation to CRC Risk That Were Reported From Each Genome‐wide G × E Interaction Analysis^a^.
**Table S8.** General Characteristics and Main Findings of the Systematic Reviews of Observational Studies. Suggestive Associations for G × E Interactions in Relation to CRC Risk That Were Identified by the Authors of the Original Systematic Reviews are Shown in Bold.
