# Supplementary material for: Gene–environment interactions and colorectal cancer risk: An umbrella review of systematic reviews and meta‐analyses of observational studies
Source: Int J Cancer. 2019 Jan 16;145(9):2315–29. doi: 10.1002/ijc.32057 (PMC6767750; doi:10.1002/ijc.32057)
Supplement: Supplementary file 1 — Appendix S1: Supplementary methods [file IJC-145-2315-s001.docx]

**Supplementary methods**

**Literature search of environmental exposures and genetic variants**

We first searched meta-analyses of the association between environmental factors and colorectal cancer (CRC) risk in the World Cancer Research Fund International (WCRF)/American Institute for Cancer Research (AICR) Third Expert Report^1^, the subsequent Continuous Update Project (CUP) CRC reports^2^ and the CUP CRC Systematic Literature Review 2016^3^. For the information of environmental factors that were not available in the WCRF/AICR Third Expert Report, the CUP CRC reports or the CUP Colorectal Systematic Literature Review 2016, we performed an additional literature search in MEDLINE and abstracted relevant data as summarized and presented in **Table 2.** For the genetic variants that were not included in the literature review by Theodoratou E et al^4^, we searched the National Human Genome Research Institute–European Bioinformatics Institute catalog of genome-wide association study (GWAS)^5^ and the GWAS central database^6^ to explore the genetic main effects by identifying published associations between genetic variants and CRC risk. Additionally, for the genetic variants that were not included in these 2 databases, a literature search in MEDLINE was carried out to identify meta-analyses and large pooled studies. Then, the study with the largest number of component studies was retained for data extraction (**Table 3**). The search strategies, the Medical Subject Headings terms and the numbers of hits are presented in **Supplementary tables 3 and 4**, respectively.

**Data extraction of the main effects of environmental exposures and genetic variants**

For the environmental main effects, we recorded the first author, journal, year of publication, the examined environmental risk factors, the number of studies considered, the study-specific relative risk estimates [relative risk (RR), odds ratio (OR)] along with the corresponding confidence intervals (CIs), the number of cases and total participants in each study for each environmental risk factor and the *I^2^* that measures the heterogeneity of the included studies in each meta-analysis of candidate-based studies or genome-wide interaction analysis (**Table 2**).

For the main genetic effects that were not included in the National Human Genome Research Institute–European Bioinformatics Institute catalog of GWAS and GWAS Central, we recorded the first author, year of publication, numbers of cases and controls in discovery and replication sets, *P* value for the main effect of variants, as well as study specific estimates (OR, RR) and CI when available from the meta-analysis or pooled study with the largest number of component studies that were identified from the literature search in MEDLINE (**Table 3**).

**Statistical analysis**

For systematic reviews, we performed descriptive analyses and presented the authors’ main conclusions. For gene-environment (G × E) interactions from candidate gene- or single-nucleotide polymorphism (SNP)-based studies with a 2-sided *P* value for interaction < 0.05 (or adjusted *P* < 0.05 after accounting for multiple comparisons), or for G × E interactions reaching genome-wide significance threshold (as specified by the author of the original publication) in genome-wide G × E interaction analyses, and we followed a set of guidelines to assess the strength of the evidence (**Table 1**). All *P* values were 2-sided.

**Supplementary results**

**Main findings of systematic reviews of observational studies**

Fifteen articles^7-21^ reported systematic reviews of observational studies on 89 G × E interactions covering 22 environmental exposures (**Supplementary table 8**). Among the 89 identified systematic reviews of the G × E interactions on CRC risk, only 3 (3.4%) pairs of interaction were concluded as suggestive by the authors. These were the interactions between methylenetetrahydrofolate reductase *(MTHFR) C677T* and folate levels (or a “high-methyl diet”)^19^, *MTHFR* *C677T* and alcohol consumption^19^, and rs3024505, rs1800872 (in or close to *﻿interleukin 10*) and frequent nonsteroidal anti-inflammatory drug (NSAID) use^8^ (**Supplementary table 8**). The lowest CRC risk was found for the *MTHFR 677TT* carriers who had higher folate levels (or a “high-methyl diet”), and the *MTHFR 677TT* homozygote subjects with the highest alcohol intake had the highest cancer risk as indicated by a systematic review^19^. The interactions between folate intake, alcohol consumption and *MTHFR* *677* gene were also explored in a meta-analysis, in which the *MTHFR 677TT* genotype with high folate intake showed lower, albeit not significant, CRC risk when compared to *677CC/CT* genotype with low folate intake [OR, 0.76 (95% CI: 0.52, 1.10); *P* = 0.14]^22^.The *677CC/TT* genotype with high alcohol consumption was also non-significantly associated with an increase in CRC risk [OR, 1.34 (95% CI: 0.92, 1.95); *P* = 0.13], but an association in the opposite direction was observed for the *677TT* genotype with high alcohol consumption [OR, 0.92 (95% CI: 0.52, 1.63); *P* = 0.78]^22^ (**Supplementary table 5**). We sought to test the interactions between folate intake, alcohol consumption and the rs1801133 (C677T) variant of *MTHFR* in data from the Genetics and Epidemiology of Colorectal Cancer Consortium (GECCO)^23^. The interaction between rs1801133 and total folate intake was not statistically significant [OR, 1.01 (95% CI: 0.93, 1.10); *P* = 0.83 by random effects; P_heterogeneity_ = 0.03; n of studies = 17]. Also, no statistically significant interaction was found between rs1801133 and overall alcohol use (P = 0.78 by fixed effects; n of studies = 27). The interactions between rs3024505, rs1800872 (in or close to *interleukin 10*) and NSAID use were associated with lower CRC risk as indicated in a systematic review^8^. The interaction between rs3024505 and NSAID use was also nominally significant in the Scottish Colorectal Cancer Study (SOCCS) (2589 controls/1874 cases, n of users = 1599, OR=1.30, 95%CI = 1.04-1.64, p=0.02 for the interaction). Notably, the interaction between rs3024505 and aspirin use was also found to be nominally significant in data from the GECCO GWAS [OR, 0.78 (95% CI: 0.62, 0.95); *P* = 0.015 by random effects; P_heterogeneity_ = 0.0075; n of studies = 15], while the interaction between rs1800872 and aspirin use was not nominally significant [OR, 0.93 (95% CI: 0.80, 1.05); *P* = 0.22 by random effects; P_heterogeneity_ = 0.11; n of studies = 15].

**Main findings of meta-analyses of candidate gene- or SNP-based studies**

Two meta-analyses of candidate SNP-based studies explored potential interactions between candidate genetic variants identified from CRC GWAS and a number of environmental CRC risk factors (including gender, body mass index, alcohol consumption, smoking, NSAID use, postmenopausal hormone use, and dietary intake of calcium, fiber, folate, red meat, processed meat, fruit, and vegetables) within the GECCO and Colon Cancer Family Registry (CCFR) consortia^24, 25^. Firstly, the presence of G × E interactions between 10 identified GWAS loci and environmental CRC risk factors was investigated in a meta-analysis^25^ of 7,106 CRC cases and 9,723 controls. Secondly, possible interactions were tested between an additional 16 SNPs and environmental risk factors in a meta-analysis comprising of 9,160 cases and 9,280 controls^24^. The strongest statistical evidence for a G × E interaction was found for vegetable consumption and rs16892766, located on chromosome 8q23.3, near the eukaryotic translation initiation factor 3 subunit H (*EIF3H)* and *UTP23* genes [OR, 1.88 (95% CI: 1.36, 2.59); nominal P for interaction = 1.3×10^-4^; adjusted *P* for interaction = 0.02] (**Supplementary** **table 5**)^25^. However, the interaction between vegetable consumption and rs16892766 was not statistically significant when validated in the GECCO GWAS [OR, 0.94 (95% CI: 0.84, 1.05); *P* = 0.25 by random effects; P_heterogeneity_ = 0.11; n of studies = 16]. No other G × E interactions were with adjusted *P* for interaction < 0.05 after accounting for multiple comparisons (all interaction results are presented in Supplementary table 4 in Hutter CM *et al* 2012^25^ and Supplementary table 2 in Kantor ED *et al* 2014^24^, separately).

One meta-analysis was performed to investigate interaction effects between red meat intake and *N*-acetyltransferase 2 (*NAT2)* on CRC risk in 2 populations, including 4 studies of Japanese (2,217 cases, 3,788 controls) and 3 studies of African Americans (527 cases, 4,527 controls)^26^. These 2 populations have high rates of CRC and a frequency of the *NAT2* rapid phenotype which is 10- and 2-fold greater than in whites, respectively^26^. The *P* of interaction between processed meat intake and *NAT2* and total meat intake and *NAT2* on CRC risk in both groups combined were 0.006 and 0.03, respectively^26^. In contrast, no significant interaction was found between red meat intake and *NAT2* in CRC in a meta-analysis of 11 studies (8,290 cases and 9,115 controls), which also included one study from Wang et al. ^27^ (**Supplementary** **table 5**). In an analysis combining individual level data from 11 studies including 8,290 cases and 9,115 controls, no significant interactions were found between red meat and *NAT2* on either the multiplicative (P = 0.99) or additive scale (P = 0.97)^27^.

A meta-analysis of 5,043 cases and 6,311 controls from 15 studies summarized the evidence for the association between serine hydroxymethyltransferase *(SHMT1)* C1420T polymorphism and CRC risk. Seven studies were included in a subgroup analysis that comprised folate intake, including 3 low folate intakes studies (609 cases/ 735 controls) and 4 high folate intake studies (957 cases/1,504 controls) as classified by the authors of the meta-analysis. Using a homozygous model (TT versus CC), *P* for the interaction between low [OR, 0.60 (95% CI: 0.40, 0.88); *P* = 0.009; *P*_heterogeneity_ = 0.75] and high [OR, 1.22 (95% CI: 0.91, 1.63); *P* = 0.29; *P*_heterogeneity_ = 0.20] folate intake was 0.004 after Bonferroni correction^28^ (**Supplementary** **table 5**). However, the interaction between rs1979277 (at *SHMT1*) and total folate intake was not statistically significant in the GECCO GWAS [OR, 1.04 (95% CI: 0.98, 1.10); *P* = 0.17; *P*_heterogeneity_ = 0.83; n of studies = 17].

An additional meta-analysis of 840 CRC patients and 1,686 controls was performed to evaluate regular aspirin use and CRC risk according to genotypes of SNP rs6983267 (8q24) and Wnt/cadherin-associated protein β1 (*CTNNB1*) expression status in tumour tissue. The effect of aspirin was found to be confined to individuals with the protective T allele of rs6983267 [matching factors-adjusted OR for T allele, 0.83 (95% CI: 0.74, 0.94); *P*_trend_ = 0.002; *P* for interaction = 0.01]^29^ ((**Supplementary** **table 5**). However, the interactions between rs6983267, rs10505477 (in strong linkage disequilibrium with rs6983267, R^2^ >0.8) and aspirin use were not statistically significant in the GECCO GWAS (P for interaction = 0.80 and 0.59 by random effects, separately).

**Main findings of genome-wide investigation of G × E interactions within GWAS consortia**

Within the GECCO/CCFR consortium, multiplicative interactions were explored between approximately 2.7 million genetic variants and red meat, processed meat, vegetables, fruit, total fiber and calcium for CRC risk^30, 31^ (**Supplementary tables 6 and 7)**. No interactions with *P* for interaction < 5.0 × 10^-8^ were observed between the examined SNPs and fruits, vegetables, fiber and calcium (total, dietary or supplemental) intake. Two interactions with *P* for interaction < 5.0 × 10^-8^ were found: the interaction between processed meat and rs4143094 at the 10p14, near GATA binding protein 3 (*GATA3*) [OR, 1.17 (95% CI: 1.11, 1.23); *P* for interaction = 8.73 × 10^-9^; *P_heterogeneity_* = 0.78], and the interaction between processed meat and rs485411 [OR, 1.18 (95% CI: 1.11, 1.25); *P* for interaction = 1.72 × 10^-8^; *P_heterogeneity_* = 0.70]^30^ (**Supplementary table 7**).

A genome-wide interaction analysis was performed to identify SNPs that modify the associations between alcohol consumption, smoking and CRC risk using data from 14 studies, totaling 8,058 cases and 8,765 controls (**Supplementary tables 6 and 7**). Using conventional logistic regression, interactions with *P* < 5.0 × 10^-8^ were found between 11 correlated SNPs (linkage disequilibrium r^2^ > 0.8) at the 9q22.32/ Hippocampus Abundant Transcript-Like 1 (*HIATL1)* locus and light-to-moderate alcohol drinking (1-28 g/day or < 2 standard drinks per day) with no evidence of heterogeneity across studies (*P_heterogeneity_* > 0.5 for any of the 11 SNPs)^32^. The rs9409565 variant had the strongest interaction effect with a *P* for interaction = 1.76 × 10^-8^ (permuted *P* = 3.51 × 10^-8^)^32^ (**Supplementary table 7**). No interactions with *P* for interaction < 5.0 × 10^-8^ were observed for SNP × smoking (smoking history and pack-years of smoking)^32^.

A study investigated multiplicative interactions between regular use of aspirin, NSAIDs, or both and approximately 2.7 million SNPs on CRC risk, comprising of 8,634 cases and 8,553 controls within the GECCO/CCFR consortium, using 2 analytical approaches (**Supplementary tables 6 and 7**). First, in logistic regression analysis of cases compared with controls, an interaction with a *P* for interaction < 5.0 × 10^-8^ was detected between aspirin and/or NSAID use and rs2965667 at the 12p12.3, near microsomal glutathione S-transferase 1 (*MGST1*) (*P* for interaction = 4.6 × 10^-9^). Second, in a case-only analysis, an interaction was found between aspirin and/or NSAID use and rs16973225 (15q25.2/ near *interleukin 16*) (*P* for interaction = 8.2 × 10^-9^)^33^ (**Supplementary table 7**).

Moreover, a genome-wide G × E search was performed to investigate whether the 3 environmental factors – NSAID usage, smoking and postmenopausal hormone use – modify the association between CRC and 7,600 genes by applying 5 gene-based methods to the whole-genome genotyped data of 10,446 cases and 10,191 controls in from the GECCO consortium (**Supplementary tables 6 and 7**). Two novel interactions were found that reached genome-wide significance level (6.6 × 10^-6^ = 0.05/7,600): the interaction between NSAIDs and patched domain containing 3 (*PTCHD3)* at 10p12.1 (including 8 variants) (*P* for interaction = 2.13 × 10^-7^ using an enhanced set-based G × E testing), and the interaction between NSAIDs and misshapen like kinase 1 (*MINK1)* at 17p13.2 (including 4 variants) (*P* for interaction = 5.65 × 10^-6^ using a case-only version of SNP-set Kernal Association Test)^34^ (**Supplementary table 7**).

A genome-wide analysis was carried out to identify genetic modifiers of CRC risk associated with use of menopausal hormone therapy, including 10,835 postmenopausal women (5,419 cases and 5,416 controls) from 10 studies (**Supplementary tables 6 and 7**). Using an empirical Bayes test, the interaction between rs964293 at 20q13.2/ cytochrome P450 family 24 sub-family A member 1 (*CYP24A1)* and use of oestrogen plus progestogen therapy was found with a *P* for interaction < 5.0 × 10^-8^ [OR, 0.61 (95% CI: 0.52, 0.72); *P* for interaction = 4.8 × 10^-9^; *P_heterogeneity_* = 0.044] (**Supplementary table 7)**.

An additional study investigated possible interactions between SNPs and 3 major environmental CRC risk factors (overweight, smoking and alcohol consumption) by adopting a 2-tiered approach comprising a case-only screening (stage I) (314 cases) and case-control validation (stage II) (259 cases, 1,002 controls) (**Supplementary tables 6 and 7**). Interactions with the smallest *P* value in stage I were verified in stage II by multiple logistic regression analysis adjusted for sex and age. Only the interaction between rs1944511 (11q23.3) and overweight passed the multiple-test correction threshold in the sex- and age-adjusted analysis of the stage II data (*P* = 0.042)^35^ (**Supplementary table 7**).

Lastly, a systematic search for G × E interactions was conducted between genotypes and 14 environmental factors by using genome-wide data from the CCFR that included 1,191 cases of microsatellite stable or microsatellite instability-low CRC and 999 controls genotyped using either the Illumina Human1M or Human1M-Duo BeadChip^36^. No G × E interactions were identified that reached genome-wide significance level of 6.5 × 10^-8^ (**Supplementary tables 6 and 7)**.

**References**

1. World Cancer Research Fund/American Institute for Cancer Research. Diet, Nutrition, Physical Activity and Cancer: a Global Perspective 2018. https://www.wcrf.org/dietandcancer.

2. World Cancer Research Fund/American Institute for Cancer Research. Continuous Update Project Report: Diet, Nutrition, Physical Activity and Colorectal Cancer, 2017. https://www.wcrf.org/sites/default/files/Colorectal-Cancer-2017-Report.pdf.

3. World Cancer Research Fund International Systematic Literature Review. The Associations between Food, Nutrition and Physical Activity and the Risk of Colorectal Cancer, 2016. https://www.wcrf.org/sites/default/files/CUP_colorectal_cancer_SLR_2016lo.pdf.

4. Theodoratou E, Timofeeva M, Li X, Meng X, Ioannidis JPA. Nature, Nurture, and Cancer Risks: Genetic and Nutritional Contributions to Cancer. *Annual review of nutrition* 2017;**37**: 293-320.

5. Welter D, MacArthur J, Morales J, Burdett T, Hall P, Junkins H, Klemm A, Flicek P, Manolio T, Hindorff L, Parkinson H. The NHGRI GWAS Catalog, a curated resource of SNP-trait associations. *Nucleic acids research* 2014;**42**: D1001-6.

6. Beck T, Hastings RK, Gollapudi S, Free RC, Brookes AJ. GWAS Central: a comprehensive resource for the comparison and interrogation of genome-wide association studies. *European journal of human genetics : EJHG* 2014;**22**: 949-52.

7. Andersen V, Holst R, Vogel U. Systematic review: diet-gene interactions and the risk of colorectal cancer. *Aliment Pharmacol Ther* 2013;**37**: 383-91.

8. Andersen V, Vogel U. Systematic review: interactions between aspirin, and other nonsteroidal anti-inflammatory drugs, and polymorphisms in relation to colorectal cancer. *Aliment Pharmacol Ther* 2014;**40**: 147-59.

9. Andersen V, Vogel U. Interactions between meat intake and genetic variation in relation to colorectal cancer. *Genes and Nutrition* 2015;**10**.

10. Corella D, Ordovas JM. Interactions between dietary n-3 fatty acids and genetic variants and risk of disease. *British Journal of Nutrition* 2012;**107**: S271-S83.

11. Cornelis MC. Gene-Coffee Interactions and Health. *Current Nutrition Reports* 2014;**3**: 178-95.

12. Cross JT, Poole EM, Ulrich CM. A review of gene-drug interactions for nonsteroidal anti-inflammatory drug use in preventing colorectal neoplasia. *Pharmacogenomics J* 2008;**8**: 237-47.

13. Eichholzer M, Luthy J, Moser U, Fowler B. Folate and the risk of colorectal, breast and cervix cancer: The epidemiological evidence. *Swiss Medical Weekly* 2001;**131**: 539-49.

14. Houlston RS, Tomlinson IP. Polymorphisms and colorectal tumor risk. *Gastroenterology* 2001;**121**: 282-301.

15. Karahalil B, Bohr VA, Wilson DM, 3rd. Impact of DNA polymorphisms in key DNA base excision repair proteins on cancer risk. *Hum Exp Toxicol* 2012;**31**: 981-1005.

16. Klarich DS, Brasser SM, Hong MY. Moderate Alcohol Consumption and Colorectal Cancer Risk. *Alcohol Clin Exp Res* 2015;**39**: 1280-91.

17. Kostner K, Denzer N, Muller CS, Klein R, Tilgen W, Reichrath J. The relevance of vitamin D receptor (VDR) gene polymorphisms for cancer: a review of the literature. *Anticancer Res* 2009;**29**: 3511-36.

18. Lenihan-Geels G, Bishop KS, Ferguson LR. Cancer risk and eicosanoid production: Interaction between the protective effect of long chain Omega-3 polyunsaturated fatty acid intake and genotype. *Journal of Clinical Medicine* 2016;**5 (2) (no pagination)**.

19. Sharp L, Little J. Polymorphisms in genes involved in folate metabolism and colorectal neoplasia: a HuGE review. *Am J Epidemiol* 2004;**159**: 423-43.

20. Shin A, Kim J. Effect modification of meat intake by genetic polymorphisms on colorectal neoplasia susceptibility. *Asian Pac J Cancer Prev* 2010;**11**: 281-7.

21. Simonds NI, Ghazarian AA, Pimentel CB, Schully SD, Ellison GL, Gillanders EM, Mechanic LE. Review of the Gene-Environment Interaction Literature in Cancer: What Do We Know? *Genetic Epidemiology* 2016;**40**: 356-65.

22. 孙丹凤, 王霞, 房静远, Xia W, Jingyuan F. 亚甲基四氢叶酸还原酶基因多态性与结肠癌发生关系的荟萃分析. *胃肠病学* 2006;**11**: 516-21.

23. Peters U, Jiao S, Schumacher FR, Hutter CM, Aragaki AK, Baron JA, Berndt SI, Bezieau S, Brenner H, Butterbach K, Caan BJ, Campbell PT, et al. Identification of Genetic Susceptibility Loci for Colorectal Tumors in a Genome-Wide Meta-analysis. *Gastroenterology* 2013;**144**: 799-807.e24.

24. Kantor ED, Hutter CM, Minnier J, Berndt SI, Brenner H, Caan BJ, Campbell PT, Carlson CS, Casey G, Chan AT, Chang-Claude J, Chanock SJ, et al. Gene-environment interaction involving recently identified colorectal cancer susceptibility Loci. *Cancer Epidemiol Biomarkers Prev* 2014;**23**: 1824-33.

25. Hutter CM, Chang-Claude J, Slattery ML, Pflugeisen BM, Lin Y, Duggan D, Nan H, Lemire M, Rangrej J, Figueiredo JC, Jiao S, Harrison TA, et al. Characterization of gene-environment interactions for colorectal cancer susceptibility loci. *Cancer Res* 2012;**72**: 2036-44.

26. Wang H, Iwasaki M, Haiman CA, Kono S, Wilkens LR, Keku TO, Berndt SI, Tsugane S, Le Marchand L. Interaction between Red Meat Intake and NAT2 Genotype in Increasing the Risk of Colorectal Cancer in Japanese and African Americans. *PLoS ONE* 2015;**10**: e0144955.

27. Ananthakrishnan AN, Du M, Berndt SI, Brenner H, Caan BJ, Casey G, Chang-Claude J, Duggan D, Fuchs CS, Gallinger S, Giovannucci EL, Harrison TA, et al. Red meat intake, NAT2, and risk of colorectal cancer: a pooled analysis of 11 studies. *Cancer Epidemiol Biomarkers Prev* 2015;**24**: 198-205.

28. Pabalan N, Jarjanazi H, Ozcelik H. A meta-analysis of the C1420T polymorphism in cytosolic serine hydroxymethyltransferase (SHMT1) among Caucasian colorectal cancer populations. *Int J Colorectal Dis* 2013;**28**: 925-32.

29. Nan H, Morikawa T, Suuriniemi M, Imamura Y, Werner L, Kuchiba A, Yamauchi M, Hunter DJ, Kraft P, Giovannucci EL, Fuchs CS, Ogino S, et al. Aspirin use, 8q24 single nucleotide polymorphism rs6983267, and colorectal cancer according to CTNNB1 alterations. *J Natl Cancer Inst* 2013;**105**: 1852-61.

30. Figueiredo JC, Hsu L, Hutter CM, Lin Y, Campbell PT, Baron JA, Berndt SI, Jiao S, Casey G, Fortini B, Chan AT, Cotterchio M, et al. Genome-wide diet-gene interaction analyses for risk of colorectal cancer. *PLoS Genet* 2014;**10**: e1004228.

31. Du M, Zhang X, Hoffmeister M, Schoen RE, Baron JA, Berndt SI, Brenner H, Carlson CS, Casey G, Chan AT, Curtis KR, Duggan D, et al. No evidence of gene-calcium interactions from genome-wide analysis of colorectal cancer risk. *Cancer Epidemiol Biomarkers Prev* 2014;**23**: 2971-6.

32. Gong J, Hutter C, Chang-Claude J, Newcomb P, Berndt S, Brenner H, Chan AT, Marchand LL, Harrison T, Lin Y, Slattery ML, White E, et al. Genome-wide interaction analyses between genetic variants and alcohol consumption and risk of colorectal cancer. *Cancer Research Conference: 105th Annual Meeting of the American Association for Cancer Research, AACR* 2014;**74**.

33. Nan H, Hutter CM, Lin Y, Jacobs EJ, Ulrich CM, White E, Baron JA, Berndt SI, Brenner H, Butterbach K, Caan BJ, Campbell PT, et al. Association of aspirin and NSAID use with risk of colorectal cancer according to genetic variants. *Jama* 2015;**313**: 1133-42.

34. Jiao S, Peters U, Berndt S, Bezieau S, Brenner H, Campbell PT, Chan AT, Chang-Claude J, Lemire M, Newcomb PA, Potter JD, Slattery ML, et al. Powerful Set-Based Gene-Environment Interaction Testing Framework for Complex Diseases. *Genetic Epidemiology* 2015;**39**: 609-18.

35. Siegert S, Hampe J, Schafmayer C, von Schonfels W, Egberts JH, Forsti A, Chen B, Lascorz J, Hemminki K, Franke A, Nothnagel M, Nothlings U, et al. Genome-wide investigation of gene-environment interactions in colorectal cancer. *Human genetics* 2013;**132**: 219-31.

36. Figueiredo JC, Lewinger JP, Song C, Campbell PT, Conti DV, Edlund CK, Duggan DJ, Rangrej J, Lemire M, Hudson T, Zanke B, Cotterchio M, et al. Genotype-environment interactions in microsatellite stable/microsatellite instability-low colorectal cancer: results from a genome-wide association study. *Cancer Epidemiol Biomarkers Prev* 2011;**20**: 758-66.
