# Supplementary material for: Gene–environment interactions and colorectal cancer risk: An umbrella review of systematic reviews and meta‐analyses of observational studies
Source: Int J Cancer. 2019 Jan 16;145(9):2315–29. doi: 10.1002/ijc.32057 (PMC6767750; doi:10.1002/ijc.32057)
Supplement: Supplementary file 2 — Figure S1 Categories for the credibility of cumulative epidemiological evidence. The 3 letters correspond (in order) to amount of evidence, replication and protection from bias. Evidence is categorized as strong, when there is A for all 3 items, and is categorized as weak when there is a C for any of the 3 items. All other combinations are categorized as moderate (from Boffetta et al 2012). Table S1. Keywords and Search Strategies for Meta‐analyses of G × E Interactions used in the Umbrella Review. Table S2. Score Categories for Credibility of an Interaction Between an Environmental Exposure and a Genetic Variant Based on the Strength of Evidence for a Main Effect of Each of Them (1 = Strong, 2 = Moderate, 3 = Weak) (adapted from Boffetta et al. 2012). Table S3. Search Strategy Used for the Identification of Main Environmental Effects for Colorectal Cancer Risk. Table S4. Search Strategy Used for the Identification of Main Genetic Effects for Colorectal Cancer Risk. Table S5. G × E Interactions in Relation to CRC Risk That Were Reported From Meta‐analyses of Candidate Gene‐ or SNP‐based Studies. Table S6. General Characteristics of 33 Genome‐wide G × E Interaction Analyses in GWAS Consortia. Table S7. SNPs With the Smallest P for G × E Interactions in Relation to CRC Risk That Were Reported From Each Genome‐wide G × E Interaction Analysisa. Table S8. General Characteristics and Main Findings of the Systematic Reviews of Observational Studies. Suggestive Associations for G × E Interactions in Relation to CRC Risk That Were Identified by the Authors of the Original Systematic Reviews are Shown in Bold. [file IJC-145-2315-s002.docx]

**Supplementary figures**

**
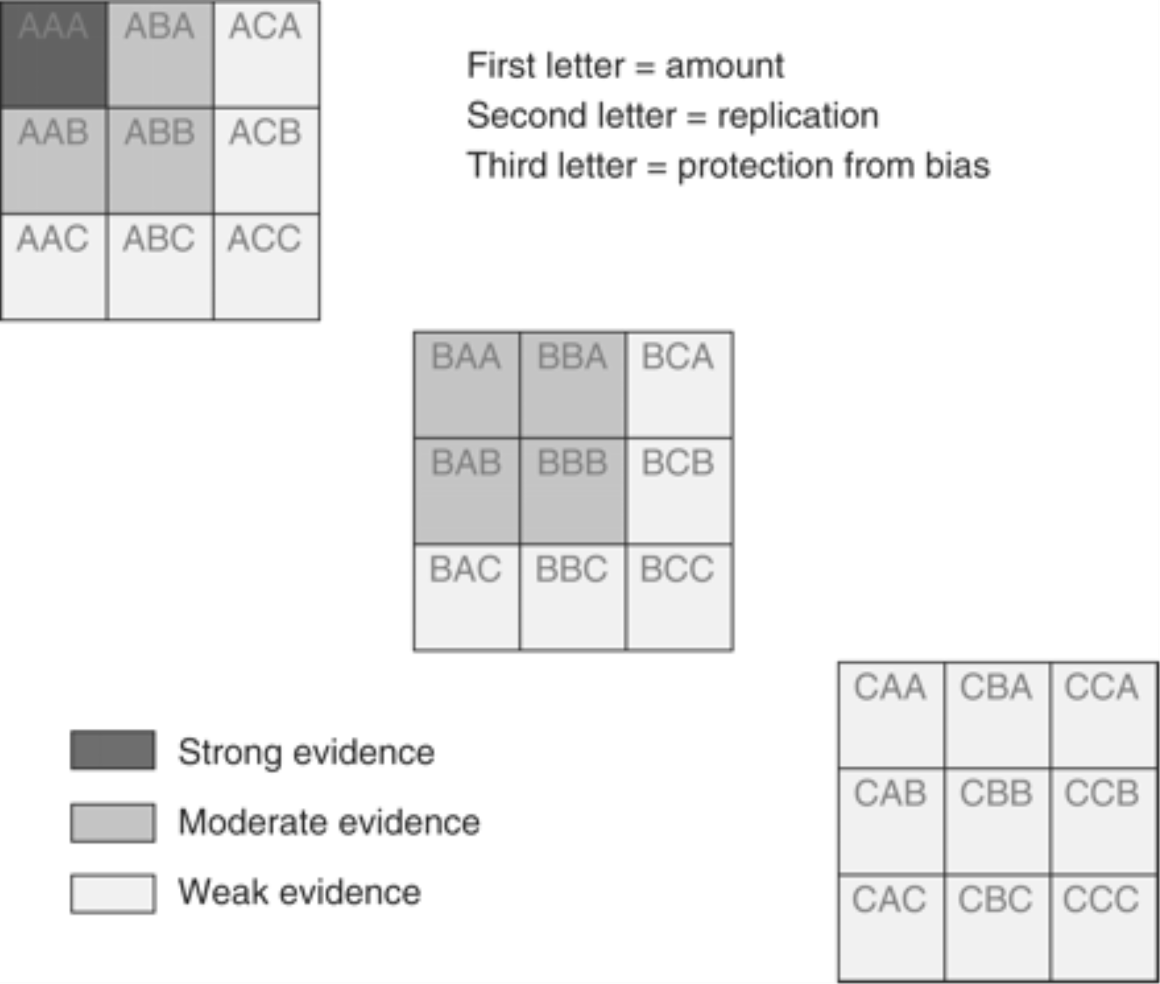
**

**Supplementary figure 1. Categories for the credibility of cumulative epidemiological evidence.** The 3 letters correspond (in order) to amount of evidence, replication and protection from bias. Evidence is categorized as strong, when there is A for all 3 items, and is categorized as weak when there is a C for any of the 3 items. All other combinations are categorized as moderate (from Boffetta et al 2012).

**Supplementary tables**

**Supplementary table 1. Keywords and Search Strategies for Meta-analyses of G × E Interactions used in the Umbrella Review.**

| **MEDLINE (OvidSP) and EMBASE (OvidSP): search strategy 1^a^** |
| --- |
| 1. colorectal cancer.mp. or exp Colorectal Neoplasms/ 2. ("colon neoplasms" or "rectal neoplasms" or ("colon" and "neoplasms") or ("rectal" and "neoplasms") or "colon neoplasms" or ("colon" and "cancer") or ("rectal" and "cancer") or "colon cancer" or "rectal cancer" or "colorectal cancer" or ("colorectal" and "cancer") or "bowel cancer" or ("bowel" and "cancer")).mp 3. 1 or 2 4. genotype environment interaction.mp. or exp genotype environment interaction/ 5. interact*.mp. 6. (G+E or GxE or gene-environment* or geno* environment*).mp 7. 4 or 5 or 6 8. ((gene* or genom*) and (infect* or inflammat* or drug* or treatment or exogenous hormon* or endogenous hormon* or chemical environment* or physical environment* or lifestyle or energy balance or method* or general or social environment* or social determin* or behav* or bacteri* or virus* or medication or personal or diet* or physical activit* or physical inactivit* or weight or obes* or body mass index or BMI or alcohol* or smoking or tobacco or carcinogens or chemical* or solvents or dioxins or pesticid* or metal* or vinyl chloride or benzidine or diesel exhaust particles or polycyclic aromatic hydrocarbons or PAH or phthalates or polychlorinated biphenyl or PCB or phenols or bisphenol A or BPA or perfluorinated compounds or perfluoro-octanoic acid or PFOA or phytoestrogens or enterolactone or ENL or genistein or cotinine or polybrominated diphenyl ether or PBDE or organochlorine pesticid* or radia* or electromagnetic field or EMF or nutri* or vitamin* or environment*)).mp 9. 3 and 7 and 8 |
| **MEDLINE (OvidSP): search strategy 2^a^** |
| 1. colorectal cancer.mp. or exp Colorectal Neoplasms/ 2. ("colon neoplasms" or "rectal neoplasms" or ("colon" and "neoplasms") or ("rectal" and "neoplasms") or "colon neoplasms" or ("colon" and "cancer") or ("rectal" and "cancer") or "colon cancer" or "rectal cancer" or "colorectal cancer" or ("colorectal" and "cancer") or "bowel cancer" or ("bowel" and "cancer")).mp 3. 1 or 2 4. genotype environment interaction.mp. or exp genotype environment interaction/ 5. interact*.mp. 6. (G+E or GxE or gene-environment* or geno* environment*).mp 7. ((gene* or genom*) and (infect* or inflammat* or drug* or treatment or exogenous hormon* or endogenous hormon* or chemical environment* or physical environment* or lifestyle or energy balance or method* or general or social environment* or social determin* or behav* or bacteri* or virus* or medication or personal or diet* or physical activit* or physical inactivit* or weight or obes* or body mass index or BMI or alcohol* or smoking or tobacco or carcinogens or chemical* or solvents or dioxins or pesticid* or metal* or vinyl chloride or benzidine or diesel exhaust particles or polycyclic aromatic hydrocarbons or PAH or phthalates or polychlorinated biphenyl or PCB or phenols or bisphenol A or BPA or perfluorinated compounds or perfluoro-octanoic acid or PFOA or phytoestrogens or enterolactone or ENL or genistein or cotinine or polybrominated diphenyl ether or PBDE or organochlorine pesticid* or radia* or electromagnetic field or EMF or nutri* or vitamin* or environment*)).mp 8. 5 and 7 9. 4 or 5 or 6 or 8 10. systematic review*.mp. or exp "Review Literature as Topic"/ 11. exp Meta-Analysis as Topic/ or meta-analys*.mp. 12. pooled.mp. 13. 10 or 11 or 12 14. 3 and 9 15. 13 and 14 |
| **EMBASE (OvidSP): search strategy 2^a^** |
| 1. colorectal cancer.mp. or exp Colorectal Neoplasms/ 2. ("colon neoplasms" or "rectal neoplasms" or ("colon" and "neoplasms") or ("rectal" and "neoplasms") or "colon neoplasms" or ("colon" and "cancer") or ("rectal" and "cancer") or "colon cancer" or "rectal cancer" or "colorectal cancer" or ("colorectal" and "cancer") or "bowel cancer" or ("bowel" and "cancer")).mp 3. 1 or 2 4. genotype environment interaction.mp. or exp genotype environment interaction/ 5. interact*.mp. 6. (G+E or GxE or gene-environment* or geno* environment*).mp 7. ((gene* or genom*) and (infect* or inflammat* or drug* or treatment or exogenous hormon* or endogenous hormon* or chemical environment* or physical environment* or lifestyle or energy balance or method* or general or social environment* or social determin* or behav* or bacteri* or virus* or medication or personal or diet* or physical activit* or physical inactivit* or weight or obes* or body mass index or BMI or alcohol* or smoking or tobacco or carcinogens or chemical* or solvents or dioxins or pesticid* or metal* or vinyl chloride or benzidine or diesel exhaust particles or polycyclic aromatic hydrocarbons or PAH or phthalates or polychlorinated biphenyl or PCB or phenols or bisphenol A or BPA or perfluorinated compounds or perfluoro-octanoic acid or PFOA or phytoestrogens or enterolactone or ENL or genistein or cotinine or polybrominated diphenyl ether or PBDE or organochlorine pesticid* or radia* or electromagnetic field or EMF or nutri* or vitamin* or environment*)).mp 8. 5 and 7 9. 4 or 5 or 6 or 8 10. exp "systematic review"/ or systematic review*.mp. 11. exp "systematic review"/ or meta-analys*.mp. 12. pooled.mp. 13. 10 or 11 or 12 14. 3 and 9 15. 13 and 14 |
| **CNKI: search strategy 1^b^** |
| (SU=“肠癌” OR SU=“肠肿瘤”) AND (SU=“基因” AND (SU=“环境” OR SU=“物理” OR SU=“化学” OR SU=“社会” OR SU=“感染” OR SU=“炎症” OR SU=“药物” OR SU=“治疗” OR SU=“激素” OR SU=“生活” OR SU=“能量” OR SU=“行为” OR SU=“微生物” OR SU=“饮食” OR SU=“活动” OR SU=“身高” OR SU=“体重” OR SU=“饮酒” OR SU=“吸烟” OR SU=“有机” OR SU=“农药” OR SU=“辐射” OR SU=“微粒” OR SU=“染料” OR SU=“营养” OR SU=“食物”))  (TI=“肠癌” OR TI=“肠肿瘤”) AND (TI=“基因” AND (TI=“环境” OR TI=“物理” OR TI=“化学” OR TI=“社会” OR TI=“感染” OR TI=“炎症” OR TI=“药物” OR TI=“治疗” OR TI=“激素” OR TI=“生活” OR TI=“能量” OR TI=“行为” OR TI=“微生物” OR TI=“饮食” OR TI=“活动” OR TI=“身高” OR TI=“体重” OR TI=“饮酒” OR TI=“吸烟” OR TI=“有机” OR TI=“农药” OR TI=“辐射” OR TI=“微粒” OR TI=“染料” OR TI=“营养” OR TI=“食物”))  (KY=“肠癌” OR KY=“肠肿瘤”) AND (KY=“基因” AND (KY=“环境” OR KY=“物理” OR KY=“化学” OR KY=“社会” OR KY=“感染” OR KY=“炎症” OR KY=“药物” OR KY=“治疗” OR KY=“激素” OR KY=“生活” OR KY=“能量” OR KY=“行为” OR KY=“微生物” OR KY=“饮食” OR KY=“活动” OR KY=“身高” OR KY=“体重” OR KY=“饮酒” OR KY=“吸烟” OR KY=“有机” OR KY=“农药” OR KY=“辐射” OR KY=“微粒” OR KY=“染料” OR KY=“营养” OR KY=“食物”))  (AB=“肠癌” OR AB=“肠肿瘤”) AND (AB=“基因” AND (AB=“环境” OR AB=“物理” OR AB=“化学” OR AB=“社会” OR AB=“感染” OR AB=“炎症” OR AB=“药物” OR AB=“治疗” OR AB=“激素” OR AB=“生活” OR AB=“能量” OR AB=“行为” OR AB=“微生物” OR AB=“饮食” OR AB=“活动” OR AB=“身高” OR AB=“体重” OR AB=“饮酒” OR AB=“吸烟” OR AB=“有机” OR AB=“农药” OR AB=“辐射” OR AB=“微粒” OR AB=“染料” OR AB=“营养” OR AB=“食物”)) |
| **Wanfang: search strategy 1^b^** |
| (“肠癌” OR “肠肿瘤”) AND (“基因” AND (“环境” OR “物理” OR “化学” OR “社会” OR “感染” OR “炎症” OR “药物” OR “治疗” OR “激素” OR “生活” OR “能量” OR “行为” OR “微生物” OR “饮食” OR “活动” OR “身高” OR “体重” OR “饮酒” OR “吸烟” OR “有机” OR “农药” OR “营养” OR “食物” OR “BMI” OR “体力”)) AND (“交互” OR “相互” OR “网络” OR “互作” OR “作用” OR “双演化” OR “交互” OR “互补”) |
| **CNKI: search strategy 2^b^** |
| (SU=“肠癌” OR SU=“肠肿瘤”) AND (SU=“基因” OR SU=“遗传” OR SU=“易感”) AND (SU=“系统综述” OR SU=“荟萃” OR SU=“meta”)  (TI=“肠癌” OR TI=“肠肿瘤”) AND (TI=“基因” OR TI=“遗传” OR TI=“易感”) AND (TI=“系统综述” OR TI=“荟萃” OR TI=“meta”)  (KY=“肠癌” OR KY=“肠肿瘤”) AND (KY=“基因” OR KY=“遗传” OR KY=“易感”) AND (KY=“系统综述” OR KY=“荟萃” OR KY=“meta”)  (AB=“肠癌” OR AB=“肠肿瘤”) AND (AB=“基因” OR AB=“遗传” OR AB=“易感”) AND (AB=“系统综述” OR AB=“荟萃” OR AB=“meta”) |
| **Wanfang: search strategy 2^b^** |
| (“肠癌” OR “肠肿瘤”) AND (“基因” OR “遗传” OR “易感”) AND (“交互” OR “相互” OR “网络” OR “互作” OR “作用” OR “双演化” OR “交互” OR “互补”) AND (“系统综述” OR “荟萃” OR “meta”) |

Abbreviation: CNKI, China National Knowledge Infrastructure; G × E, gene-environment; EMBASE, Excerpta Medica Database; MEDLINE, Medical Literature Analysis and Retrieval System.

^a^ For MEDLINE and EMBASE, we used both AND and OR to combine the keywords “G × E interactions” and ((gene* OR genom*) AND specific environmental risk factors), considering that there might be some publications that did not include the keyword “G × E interactions”.

^b^ For CNKI and Wanfang, both strategies that included and not included specific environmental risk factors were used due to the limit of length of search strategies in these 2 databases.

**Supplementary table 2. Score Categories for Credibility of an Interaction Between an Environmental Exposure and a Genetic Variant Based on the Strength of Evidence for a Main Effect of Each of Them (1 = Strong, 2 = Moderate, 3 = Weak) (adapted from Boffetta et al. 2012).**

|  | **Evidence for environmental main effect** | | | |
| --- | --- | --- | --- | --- |
| **Evidence for genetic main effect** | I (Convincing) | II (Highly suggestive) | III (Suggestive) | IV (Weak) |
| Strong | 1 | 2 | 2 | 3 |
| Moderate | 2 | 2 | 3 | 3 |
| Weak | 2 | 3 | 3 | 3 |
| Lack/evidence against | 3 | 3 | 3 | 3 |

**Supplementary table 3. Search Strategy Used for the Identification of Main Environmental Effects for Colorectal Cancer Risk.**

| **Environmental factor** | **Search strategy** | **Date** | **Number of hits** |
| --- | --- | --- | --- |
| Oestrogen plus progestogen therapy | ((colorectal cancer.mp. or exp Colorectal Neoplasms/) or (("colon neoplasms" or "rectal neoplasms" or ("colon" and "neoplasms") or ("rectal" and "neoplasms") or "colon neoplasms" or ("colon" and "cancer") or ("rectal" and "cancer") or "colon cancer" or "rectal cancer" or "colorectal cancer" or ("colorectal" and "cancer") or "bowel cancer" or ("bowel" and "cancer")).mp.)) and (Estrogen Replacement Therapy.mp. or exp Estrogen Replacement Therapy/ or menopausal hormone therapy.mp. or oestrogen progestogen therapy.mp.) | 10/10/2018 | 322 |
| Aspirin | ((colorectal cancer.mp. or exp Colorectal Neoplasms/) or (("colon neoplasms" or "rectal neoplasms" or ("colon" and "neoplasms") or ("rectal" and "neoplasms") or "colon neoplasms" or ("colon" and "cancer") or ("rectal" and "cancer") or "colon cancer" or "rectal cancer" or "colorectal cancer" or ("colorectal" and "cancer") or "bowel cancer" or ("bowel" and "cancer")).mp.)) and (aspirin.mp. or Aspirin/ or acetylsalicylic.mp. or salicylic acid.mp. or Salicylic Acid/) | 10/10/2018 | 1,526 |
| Aspirin and/or nonsteroidal anti-inflammatory drug use | ((colorectal cancer.mp. or exp Colorectal Neoplasms/) or (("colon neoplasms" or "rectal neoplasms" or ("colon" and "neoplasms") or ("rectal" and "neoplasms") or "colon neoplasms" or ("colon" and "cancer") or ("rectal" and "cancer") or "colon cancer" or "rectal cancer" or "colorectal cancer" or ("colorectal" and "cancer") or "bowel cancer" or ("bowel" and "cancer")).mp.)) and (aspirin.mp. or Aspirin/ or acetylsalicylic.mp. or salicylic acid.mp. or Salicylic Acid/ or anti-inflammatory.mp. or Anti-Inflammatory Agents/ or Nonsteroidal Anti-Androgens/ or nonsteroidal.mp. or NSAID.mp. or Anti-Inflammatory Agents, Non-Steroidal/) | 10/10/2018 | 4,389 |

**Supplementary table 4. Search Strategy Used for the Identification of Main Genetic Effects for Colorectal Cancer Risk.**

| **Genetic variant** | **Search strategy** | **Date** | **Number of hits** |
| --- | --- | --- | --- |
| rs9409565 (*HIATL1*) | ((colorectal cancer.mp. or exp Colorectal Neoplasms/) or (("colon neoplasms" or "rectal neoplasms" or ("colon" and "neoplasms") or ("rectal" and "neoplasms") or "colon neoplasms" or ("colon" and "cancer") or ("rectal" and "cancer") or "colon cancer" or "rectal cancer" or "colorectal cancer" or ("colorectal" and "cancer") or "bowel cancer" or ("bowel" and "cancer")).mp.)) and (*HIATL1*.mp. or rs9409565.mp or 9q22.mp.) | 14/03/2017 | 11 |
| *NAT2* | ((colorectal cancer.mp. or exp Colorectal Neoplasms/) or (("colon neoplasms" or "rectal neoplasms" or ("colon" and "neoplasms") or ("rectal" and "neoplasms") or "colon neoplasms" or ("colon" and "cancer") or ("rectal" and "cancer") or "colon cancer" or "rectal cancer" or "colorectal cancer" or ("colorectal" and "cancer") or "bowel cancer" or ("bowel" and "cancer")).mp.)) and ((Arylamine N-Acetyltransferase/ or NAT2.mp. or Arylamine N-Acetyltransferase/ or N-Acetyltransferase 2.mp.)) and (systematic review*.mp. or Review Literature.mp. or "Review" or Meta-Analysis as Topic or meta-analys*.mp. or pooled.mp.) | 25/11/2017 | 50 |
| C1420T *(SHMT1)* | ((colorectal cancer.mp. or exp Colorectal Neoplasms/) or (("colon neoplasms" or "rectal neoplasms" or ("colon" and "neoplasms") or ("rectal" and "neoplasms") or "colon neoplasms" or ("colon" and "cancer") or ("rectal" and "cancer") or "colon cancer" or "rectal cancer" or "colorectal cancer" or ("colorectal" and "cancer") or "bowel cancer" or ("bowel" and "cancer")).mp.)) and (C1420T.mp. or SHMT1.mp. or serine hydroxymethyltransferase.mp. or exp Glycine Hydroxymethyltransferase/) | 01/11/2017 | 25 |
| rs964293 *(CYP24A1)* | ((colorectal cancer.mp. or exp Colorectal Neoplasms/) or (("colon neoplasms" or "rectal neoplasms" or ("colon" and "neoplasms") or ("rectal" and "neoplasms") or "colon neoplasms" or ("colon" and "cancer") or ("rectal" and "cancer") or "colon cancer" or "rectal cancer" or "colorectal cancer" or ("colorectal" and "cancer") or "bowel cancer" or ("bowel" and "cancer")).mp.)) and (rs964293.mp. or *CYP24A1.*mp.) | 01/11/2017 | 49 |
| *PTCHD3* | PTCHD3.mp. | 01/11/2017 | 10 |
| *MINK1* | Misshapen-like kinase 1.mp. or MINK1.mp. | 01/11/2017 | 22 |
| rs2965667 *(PIK3C2G)* | ((colorectal cancer.mp. or exp Colorectal Neoplasms/) or (("colon neoplasms" or "rectal neoplasms" or ("colon" and "neoplasms") or ("rectal" and "neoplasms") or "colon neoplasms" or ("colon" and "cancer") or ("rectal" and "cancer") or "colon cancer" or "rectal cancer" or "colorectal cancer" or ("colorectal" and "cancer") or "bowel cancer" or ("bowel" and "cancer")).mp.)) and (rs2965667.mp. or PIK3C2G.mp. or exp Phosphatidylinositol 3-Kinases/ or PIK3C2G.mp. or MGST1.mp.) | 01/11/2017 | 1,251 |
| rs16973225 *(IL6)* | ((colorectal cancer.mp. or exp Colorectal Neoplasms/) or (("colon neoplasms" or "rectal neoplasms" or ("colon" and "neoplasms") or ("rectal" and "neoplasms") or "colon neoplasms" or ("colon" and "cancer") or ("rectal" and "cancer") or "colon cancer" or "rectal cancer" or "colorectal cancer" or ("colorectal" and "cancer") or "bowel cancer" or ("bowel" and "cancer")).mp.)) and (rs16973225.mp. or IL6.mp. or Interleukin-6.mp. or exp Interleukin-6/) | 01/11/2017 | 1,006 |
| rs1944511 (11q23) | ((colorectal cancer.mp. or exp Colorectal Neoplasms/) or (("colon neoplasms" or "rectal neoplasms" or ("colon" and "neoplasms") or ("rectal" and "neoplasms") or "colon neoplasms" or ("colon" and "cancer") or ("rectal" and "cancer") or "colon cancer" or "rectal cancer" or "colorectal cancer" or ("colorectal" and "cancer") or "bowel cancer" or ("bowel" and "cancer")).mp.)) and (rs1944511.mp. or 11q23.mp.) | 22/12/2017 | 32 |

**Supplementary table 5. G × E Interactions in Relation to CRC Risk That Were Reported From Meta-analyses of Candidate Gene- or SNP-based Studies.**

| **Environmental exposure** | **Genetic variant** | **Gene (or near gene)** | **Number of studies in meta-analysis** | **Number of participants** | **Number of cases** | **Meta-analysis model** | ***P* value for interaction** | **Heterogeneity, *I^2^* or *P* value for heterogeneity** |  |
| --- | --- | --- | --- | --- | --- | --- | --- | --- | --- |
| Red meat | | | | | | | | | |
| Andersen V, 2013 | Slow/fast | *NAT1* | 2 | 2,964 | 1,221 | NA | 0.95 | NA |  |
| Andersen V, 2013 | Slow/fast | *NAT2* | 3 | 3,590 | 1,404 | NA | 0.07 | NA |  |
| Hutter CM, 2012^a^ | rs4939827 | 18q21*/SMAD7* | 9 | 16,739 | 7,016 | Fixed | Nominal *P*=2.9E-03; adjusted *P*=0.36 | *P*=0.59 |  |
| Hutter CM, 2012^a^ | rs3802842 | 11q23/*LOC120376* | 7 | 16,739 | 7,016 | Fixed | Nominal *P*=8.6E-03; adjusted *P*=0.73 | *P*=0.58 |  |
| Ananthakrishan AN, 2015 | Slow/intermediate/rapid | *NAT2* | 11 | 17,045^b^ | 8,290^b^ | NA | Multiplicative *P*=0.99; additive *P*=0.97 | NA |  |
| Wang H, 2015 | Slow/intermediate/rapid | *NAT2* | 7 | 10,744 | 2,652 | Fixed | 0.030 | *I^2^*=0% |  |
| Processed meat | | | | | | | | | |
| Wang H, 2015 | Slow/intermediate/rapid | *NAT2* | 7 | 10,744 | 2,652 | Fixed | 0.006 | *I^2^*=49.7% |  |
| Red meat without processed meat | | | | | | | | | |
| Wang H, 2015 | Slow/intermediate/rapid | *NAT2* | 7 | 10,744 | 2,652 | Fixed | 0.17 | *I^2^*=0% |  |
| Alcohol consumption | | | | | | | | | |
| Liu Y, 2011 | rs1042522  (Pro72Arg) | *p53* | 2 | NA | NA | Fixed or random | Arg/Arg: 0.11;  Pro/Arg: 0.09;  Pro/Pro: 0.73 | NA |  |
| Ding W, 2013 | rs1805087 (A2756G) | *MTR* | 4 | NA | NA | Fixed or random | *P*=0.002 (G allele carriers vs AA genotype among heavy drinkers) | *P*=0.38 (among heavy drinkers) |  |
| Sun D, 2006 |  | *MTHFR 677* | NA | 3,843^b^ | 1,608^b^ | Fixed | Low intake with TT: 0.78; High intake with CC/CT: 0.13; High intake with TT: 0.62 | NA |  |
| Sun D, 2006 |  | *MTHFR 1298* | NA | 3,237^b^ | 1,285^b^ | Fixed | Low intake with AC/CC: 0.08; High intake with AA: 0.57; High intake with AC/CC: 0.78 | NA |  |
| Hutter CM, 2010 | rs10808555 | 8q24 | 2 | 4,326 | 1,987 | NA | 0.70 | NA |  |
| Hutter CM, 2010 | rs6983267 | 8q24 | 2 | 4,326 | 1,987 | NA | 0.91 | NA |  |
| Hutter CM, 2010 | rs10956368 | 8q24 | 2 | 4,326 | 1,987 | NA | 0.95 | NA |  |
| Vegetables | | | | | | | | | |
| Hutter CM, 2012^a^ | rs16892766 | 8q23.3*/EIF3H/UTP23* | 7 | 16,739 | 7,016 | Fixed | Nominal *P*=1.3×10^-4^; adjusted *P*=0.02 | *P*=0.68 |  |
| Hutter CM, 2012^a^ | rs16892766 | 8q23.3*/EIF3H/UTP23* | 9 | 16,739 | 7,016 | Fixed | Nominal *P*=0.0035; adjusted *P*=0.40 | *P*=0.31 |  |
| Kantor ED, 2014^a^ | rs7136702 | 12q13.13/*LARP4/DIP2B* | 13 | 18,440 | 9,160 | Fixed | Nominal *P*=0.00739; adjusted *P*=0.77 | *P*=0.68 |  |
| Cruciferous vegetables | | | | | | | | | |
| Tse G, 2014 | Present/null | *GSTM1* | 8 | 12,383 | 4,016 | Random | NA | NA |  |
| Tse G, 2014 | Present/null | *GSTT1* | 6 | 11,144 | 3,556 | Random | NA | NA |  |
| Tse G, 2014 | Present/null | *GSTM1 and GSTT1* | 6 | 11,144 | 3,556 | Random | NA | NA |  |
| Fiber | | | | | | | | | |
| Hutter CM, 2012^a^ | rs16892766 | 8q23.3*/EIF3H/UTP23* | 5 | 16,739 | 7,016 | Fixed | Nominal *P*=6.0×10^-4^; adjusted *P*=0.09 | *P*=0.87 |  |
| Kantor ED, 2014^a^ | rs3217810 | 12p13.32/*CCND2* | 13 | 18,440 | 9,160 | Fixed | Nominal *P*=0.00298; adjusted *P*=0.45 | *P*=0.20 |  |
| Folate | | | | | | | | | |
| Sun D, 2006 |  | *MTHFR 677* | NA | 2,964^b^ | 1,249^b^ | Fixed | Low intake with TT: 0.09; High intake with CC/CT: 0.01; High intake with TT: 0.14 | NA |  |
| Sun D, 2006 |  | *MTHFR* 1298 | NA | 3,164^b^ | 1,278^b^ | Fixed | Low intake with AC/CC: 0.17; High intake with AA: 0.01; High intake with AC/CC: 0.02 | NA |  |
| Hutter CM, 2012^a^ | rs3802842 | 11q23/*LOC120376* | 5 | 16,739 | 7,016 | Fixed | Nominal *P*=0.0082; adjusted *P*=0.71 | *P*=0.65 |  |
| Kantor ED, 2014^a^ | rs3217810 | 12q13.13/*CCND2* | 13 | 18,440 | 9,160 | Fixed | Nominal *P*=0.00411; adjusted *P*=0.56 | *P*=0.12 |  |
| Pabalan N, 2013 | *C1420T* | *SHMT1* | 7 | 3,805 | 1,566 | Fixed | 0.004 (Bonferroni-corrected) | *P*=0.20 for the 4 high intake studies and *P*^=^0.75 for the 3 low intake studies |  |
| Fruit | | | | | | | | | |
| Kantor ED, 2014^a^ | rs719725 | 9p24*/TPD52L3/interleukin 33/UHRF2/GLDC* | 13 | 18,440 | 9,160 | Fixed | Nominal *P*=0.00959; adjusted *P*=0.86 | *P*=0.90 |  |
| Smoking | | | | | | | | | |
| Raimondi S, 2009 | Present/null | *GSTM1* | 8 | 5,413 | 2,014 | Random | 0.77 | NA |  |
| Raimondi S, 2009 | Present/null | *GSTT1* | 5 | 3,239 | 1,434 | Random | 0.94 | NA |  |
| Raimondi S, 2009 | Slow/intermediate/fast | *NAT2* | 6 | 7,688 | 3,233 | Random | 0.98 | NA |  |
| Ding W, 2013 | rs1805087 (A2756G) | *MTR* | 4 | NA | NA | Fixed or random | *P*=0.001 (G allele carriers vs AA genotype among heavy smokers) | *P*=0.29 (among heavy smokers) |  |
| Smits, KM, 2003 | Present/null | *GSTM1* | 3 | 1,160^b^ | 414^b^ | Random | χ2=0.007, *P* > 0.99, degree of freedom = 1 | NA |  |
| Hutter CM, 2010 | rs10808555 | 8q24 | 2 | 4,326 | 1,987 | NA | 0.89 | NA |  |
| Hutter CM, 2010 | rs6983267 | 8q24 | 2 | 4,326 | 1,987 | NA | 0.43 | NA |  |
| Hutter CM, 2010 | rs10956368 | 8q24 | 2 | 4,326 | 1,987 | NA | 0.82 | NA |  |
| Pabalan N, 2008 | G870A | *CCND1* | 14 of all cancer types | NA | NA | Fixed and random | NA | NA |  |
| Wan H, 2010 | Positive/null | *GSTT1* | 7 | 2,578 smokers and 2,519 non-smokers | NA | Random | Null vs positive among smokers: 0.47; Null vs positive among non-smokers: 0.94; Smokers with null vs non-smokers with positive genotype: 0.20 | NA |  |
| Wang X, 2014 | rs2234767 | *FAS* | 13 | 7,110 | 3,143 | Fixed | NA | *P*=0.104 for smokers and *P*=0.073 for non-smokers |  |
| Zhang L, 2012 | Slow/rapid | *NAT2* | 11 | NA | NA | Fixed or random | NA | *P*=0.01 for never smokers and *P*=0.07 for ever smokers |  |
| Jiao S, 2013 | A marker set comprising of 25 known CRC loci |  | Studies within GECCO consortium | 24,057 | 10,729 | NA | 0.00592 | NA |  |
| BMI | | | | | | | | | |
| Hutter CM, 2010 | rs10808555 | 8q24 | 2 | 4,326 | 1,987 | NA | 0.08 | NA |  |
| Hutter CM, 2010 | rs6983267 | 8q24 | 2 | 4,326 | 1,987 | NA | 0.62 | NA |  |
| Hutter CM, 2010 | rs10956368 | 8q24 | 2 | 4,326 | 1,987 | NA | 0.48 | NA |  |
| Physical activity | | | | | | | | | |
| Hutter CM, 2010 | rs10808555 | 8q24 | 2 | 4,326 | 1,987 | NA | 0.23 | NA |  |
| Hutter CM, 2010 | rs6983267 | 8q24 | 2 | 4,326 | 1,987 | NA | 0.66 | NA |  |
| Hutter CM, 2010 | rs10956368 | 8q24 | 2 | 4,326 | 1,987 | NA | 0.33 | NA |  |
| Regular aspirin use | | | | | | | | | |
| Nan H, 2013 | rs6983267 | 8q24 | 2 | 2,526^b^ | 840^b^ | NA | 0.01 | Consistent in each study |  |
| Dose of aspirin intake | | | | | | | | | |
| Nan H, 2013 | rs6983267 | 8q24 | 2 | 2,514^b^ | 834^b^ | NA | 0.08 | NA |  |
| Duration of regular aspirin use | | | | | | | | | |
| Nan H, 2013 | rs6983267 | 8q24 | 2 | 2,524^b^ | 839^b^ | NA | 0.20 | NA |  |
| Aspirin use in CTNNB1-positive | | | | | | | | | |
| Nan H, 2013 | rs6983267 | 8q24 | 2 | 1,748^b^ | 125^b^ | NA | 0.04 | NA |  |
| Aspirin use in CTNNB1-negative | | | | | | | | | |
| Nan H, 2013 | rs6983267 | 8q24 | 2 | 1,773^b^ | 150^b^ | NA | 0.33 | NA |  |
| NSAID use | | | | | | | | | |
| Hutter CM, 2010 | rs10808555 | 8q24 | 2 | 4,326 | 1,987 | NA | 0.16 | NA |  |
| Hutter CM, 2010 | rs6983267 | 8q24 | 2 | 4,326 | 1,987 | NA | 0.73 | NA |  |
| Hutter CM, 2010 | rs10956368 | 8q24 | 2 | 4,326 | 1,987 | NA | 0.55 | NA |  |
| Hormone Replacement Therapy use | | | | | | | | | |
| Hutter CM, 2010 | rs10808555 | 8q24 | 2 | 4,326 | 1,987 | NA | 0.88 | NA |  |
| Hutter CM, 2010 | rs6983267 | 8q24 | 2 | 4,326 | 1,987 | NA | 0.71 | NA |  |
| Hutter CM, 2010 | rs10956368 | 8q24 | 2 | 4,326 | 1,987 | NA | 0.83 | NA |  |
| Postmenopausal hormone use | | | | | | | | | |
| Kantor ED, 2014^a^ | rs6691170 | 1q41/*DUSP10* | 13 | 18,440 | 9,160 | Fixed | Nominal *P*=0.00174; adjusted *P*=0.30 | *P*=0.18 |  |
| Gender | | | | | | | | | |
| Hutter CM, 2010 | rs10808555 | 8q24 | 2 | 4,326 | 1,987 | NA | 0.45 | NA |  |
| Hutter CM, 2010 | rs6983267 | 8q24 | 2 | 4,326 | 1,987 | NA | 0.33 | NA |  |
| Hutter CM, 2010 | rs10956368 | 8q24 | 2 | 4,326 | 1,987 | NA | 0.39 | NA |  |
| Kantor ED, 2014^a^ | rs10936599 | 3q26.2/*MYNN* | 13 | 18,440 | 9,160 | Fixed | Nominal *P*=0.00773; adjusted *P*=0.78 | *P*=0.31 |  |
| Circulating 25-Hydroxyvitamin [25(OH)D] | | | | | | | | | |
| Hiraki LT, 2014 | A genetic risk score comprising of 31 CRC associated SNPs |  | 3 | 1,581^b^ | 672^b^ | Fixed | No association (*P* > 0.05) | *P*=0.98 |  |

Abbreviations: BMI, body mass index; CRC, colorectal cancer; G × E, gene-environment; NA, not available; NSAID, nonsteroidal anti-inflammatory drug; SNP, single-nucleotide polymorphisms; vs, versus.

^a^ G × E interactions with *P* value for interaction <0.01 identified from Hutter CM et al. and Kantor ED et al. All interaction results from these 2 studies are presented in Supplementary table S4 in Hutter CM et al. 2012 and in Supplementary table S2 in Kantor ED et al. 2014.

^b^ The exact numbers of participants included in the statistical analyses instead of the total number of participants from all studies.

**Supplementary table 6. General Characteristics of 33 Genome-wide G × E Interaction Analyses in GWAS Consortia.**

| **Environmental exposure** | **Genetic variant** | **Environmental exposure measurement** | **Genetic array** | **Population** | **Number of studies** | **Number of participants** | **Number of cases** | **Meta-analysis model** |
| --- | --- | --- | --- | --- | --- | --- | --- | --- |
| **Red meat** | | | | | | | | |
| Figueiredo JC, 2011 | 770,098 SNPs | Mailed questionnaires or a telephone-based or face-to-face interview | The Illumina Human1M (n individuals=1,973; m=1,072,820 SNPs) or Human1M-Duo (n individuals=374; m=1,199,187 SNPs) BeadChip platforms | Self-identified as non-Hispanic white | 3 population-based registries | 2,190 | 1,191 | NA |
| Figueiredo JC, 2014 | Approximately 2.7 million SNPs | In-person interviews and/or structured questionnaires | HapMap II | European ancestry | 10 | 18,404 | 9,287 | Fixed |
| **Processed meat** | | | | | | | | |
| Figueiredo JC, 2014 | Approximately 2.7 million SNPs | In-person interviews and/or structured questionnaires | HapMap II | European ancestry | 10 | 18,404 | 9,287 | Fixed |
| **Total fiber** | | | | | | | | |
| Figueiredo JC, 2014 | Approximately 2.7 million SNPs | In-person interviews and/or structured questionnaires | HapMap II | European ancestry | 10 | 18,404 | 9,287 | Fixed |
| **Total calcium intake (dietary + supplemental)** | | | | | | | | |
| Du M, 2014 | Approximately 2.7 million SNPs | FFQ or diet history in DALS | HapMap CEU | European ancestry | 13 | 18,509 | 9,006 | Fixed |
| **Dietary calcium intake** | | | | | | | | |
| Du M, 2014 | Approximately 2.7 million SNPs | FFQ or diet history in DALS | HapMap CEU | European ancestry | 13 | 18,509 | 9,006 | Fixed |
| **Supplementary calcium intake** | | | | | | | | |
| Figueiredo JC, 2011 | 770,098 SNPs | Mailed questionnaires or a telephone-based or face-to-face interview | The Illumina Human1M (n individuals=1,973; m=1,072,820 SNPs) or Human1M-Duo (n individuals=374; m=1,199,187 SNPs) BeadChip platforms | Non-Hispanic white | 3 population-based registries | 2,190 | 1,191 | NA |
| Du M, 2014 | Approximately 2.7 million SNPs | FFQ or diet history in DALS | HapMap CEU | European ancestry | 13 | 18,509 | 9,006 | Fixed |
| **Alcohol consumption** | | | | | | | | |
| Figueiredo JC, 2011 | 770,098 SNPs | Mailed questionnaires or a telephone-based or face-to-face interview | The Illumina Human1M (n individuals=1973; m=1,072,820 SNPs) or Human1M-Duo (n individuals=374; m=1,199,187 SNPs) BeadChip platforms | Non-Hispanic white | 3 population-based registries | 2,190 | 1,191 | NA |
| Gong J, 2016 | Approximately 2.7 million SNPs | Interviews or self-administered questionnaires | HapMap CEU | European ancestry | 14 | 16,823 | 8,058 | Fixed |
| Siegert S, 2013 | 4,373,163 SNPs in stage I | All participants completed a baseline questionnaire or interview at the time of enrollment. 76 % of cases completed a follow-up questionnaire on their body weight 1 year before CRC was diagnosed. | Affymetrix Genome-Wide Human SNP Array 500k (containing 500,568 SNPs) for the sporadic CRC samples, and Affymetrix Genome-Wide Human SNP Array 6.0 (934,968 SNPs) for the patients with a positive family history of CRC and all controls | German descent | From the PopGen biobank | 314 in case-only analysis;  1,261 in case-control analysis to validate findings of case-only analysis | 314 in case-only analysis;  259 in case-control analysis to validate findings of case-only analysis | NA |
| **Smoking** | | | | | | | | |
| Figueiredo JC, 2011 | 770,098 SNPs | Mailed questionnaires or a telephone-based or face-to-face interview | The Illumina Human1M (n individuals=1,973; m=1,072,820 SNPs) or Human1M-Duo (n individuals=374; m=1,199,187 SNPs) BeadChip platforms | Non-Hispanic white | 3 population-based registries | 2,190 | 1,191 | NA |
| Gong J, 2016 | Approximately 2.7 million SNPs | Interviews or self-administered questionnaires | HapMap CEU | European ancestry | 14 | 22,601 | 11,219 | Fixed |
| Jiao S, 2015 | 7,600 genes | Questionnaires and/or interviews | The Illumina HumanExome Beadchip data | European descent | 7 nested case-control and 5 case-control studies in the GECCO consortium | 20,637 | 10,446 | NA |
| Siegert S, 2013 | 4,373,163 SNPs in stage I | All participants completed a baseline questionnaire or interview at the time of enrollment. 76 % of cases completed a follow-up questionnaire on their body weight 1 year before CRC was diagnosed. | Affymetrix Genome-Wide Human SNP Array 500k (containing 500,568 SNPs) for the sporadic CRC samples, and Affymetrix Genome-Wide Human SNP Array 6.0 (934,968 SNPs) for the patients with a positive family history of CRC and all controls | German descent | From the PopGen biobank | 314 in stage I and 1261 in stage II | 314 in stage I and 259 in stage II | NA |
| **BMI and overweight** | | | | | | | | |
| Figueiredo JC, 2011 | 770,098 SNPs | Mailed questionnaires or a telephone-based or face-to-face interview | The Illumina Human1M (n individuals=1,973; m=1,072,820 SNPs) or Human1M-Duo (n individuals=374; m=1,199,187 SNPs) BeadChip platforms | Non-Hispanic white | 3 population-based registries | 2,190 | 1,191 | NA |
| Siegert S, 2013 | 4,373,163 SNPs in stage I | All participants completed a baseline questionnaire or interview at the time of enrollment. 76 % of cases completed a follow-up questionnaire on their body weight 1 year before CRC was diagnosed. Body mass index were calculated from self-reported weight and height data. | Affymetrix Genome-Wide Human SNP Array 500k (containing 500,568 SNPs) for the sporadic CRC samples, and Affymetrix Genome-Wide Human SNP Array 6.0 (934,968 SNPs) for the patients with a positive family history of CRC and all controls | German descent | From the PopGen biobank | 314 in stage I and 1261 in stage II | 314 in stage I and 259 in stage II | NA |
| **Folic acid use (including multivitamin users)** | | | | | | | | |
| Figueiredo JC, 2011 | 770,098 SNPs | Mailed questionnaires or a telephone-based or face-to-face interview | The Illumina Human1M (n individuals=1973; m=1072 820 SNPs) or Human1M-Duo (n individuals=374; m=1199 187 SNPs) BeadChip platforms | Non-Hispanic white | 3 population-based registries | 2,190 | 1,191 | NA |
| **Multivitamin use** | | | | | | | | |
| Figueiredo JC, 2011 | 770,098 SNPs | Mailed questionnaires or a telephone-based or face-to-face interview | The Illumina Human1M (n individuals=1,973; m=1,072,820 SNPs) or Human1M-Duo (n individuals=374; m=1,199,187 SNPs) BeadChip platforms | Non-Hispanic white | 3 population-based registries | 2,190 | 1,191 | NA |
| **Fruit** | | | | | | | | |
| Figueiredo JC, 2011 | 770,098 SNPs | Mailed questionnaires or a telephone-based or face-to-face interview | The Illumina Human1M (n individuals=1,973; m=1,072,820 SNPs) or Human1M-Duo (n individuals=374; m=1,199,187 SNPs) BeadChip platforms | Non-Hispanic white | 3 population-based registries | 2,190 | 1,191 | NA |
| Figueiredo JC, 2014 | Approximately 2.7 million SNPs | In-person interviews and/or structured questionnaires | HapMap II | European ancestry | 10 | 18,404 | 9,287 | Fixed |
| **Vegetables** | | | | | | | | |
| Figueiredo JC, 2011 | 770,098 SNPs | Mailed questionnaires or a telephone-based or face-to-face interview | The Illumina Human1M (n individuals=1,973; m=1,072,820 SNPs) or Human1M-Duo (n individuals=374; m=1,199,187 SNPs) BeadChip platforms | Non-Hispanic white | 3 population-based registries | 2,190 | 1,191 | NA |
| Figueiredo JC, 2014 | Approximately 2.7 million SNPs | In-person interviews and/or structured questionnaires | HapMap II | European ancestry | 10 | 18,404 | 9,287 | Fixed |
| **Physical activity** | | | | | | | | |
| Figueiredo JC, 2011 | 770,098 SNPs | Mailed questionnaires or a telephone-based or face-to-face interview | The Illumina Human1M (n individuals=1,973; m=1,072,820 SNPs) or Human1M-Duo (n individuals=374; m=1,199,187 SNPs) BeadChip platforms | Non-Hispanic white | 3 population-based registries | 2,190 | 1,191 | NA |
| **Height** | | | | | | | | |
| Figueiredo JC, 2011 | 770,098 SNPs | Mailed questionnaires or a telephone-based or face-to-face interview | The Illumina Human1M (n individuals=1,973; m=1,072,820 SNPs) or Human1M-Duo (n individuals=374; m=1,199,187 SNPs) BeadChip platforms | Non-Hispanic white | 3 population-based registries | 2,190 | 1,191 | NA |
| **Aspirin use** | | | | | | | | |
| Nan H, 2015 | Approximately 2.7 million SNPs | In-person interviews and/or structured questionnaires | All autosomal SNPs of each study were imputed to the CEPH collection (CEU) population in HapMap II using IMPUTE (CCFR), BEAGLE (OFCCR), and MACH (all other studies). | European descent | 5 case-control and 5 cohort studies from CCFR and GECCO consortium | 17,187 | 8,634 | Fixed |
| **Aspirin and/or NSAID use** | | | | | | | | |
| Nan H, 2015 | Approximately 2.7 million SNPs | In-person interviews and/or structured questionnaires | All autosomal SNPs of each study were imputed to the CEPH collection (CEU) population in HapMap II using IMPUTE (CCFR), BEAGLE (OFCCR), and MACH (all other studies). | European descent | 5 case-control and 5 cohort studies from CCFR and GECCO consortium | 17,187 | 8,634 | Fixed |
| Figueiredo JC, 2011 | 770,098 SNPs | Mailed questionnaires or a telephone-based or face-to-face interview | The Illumina Human1M (n individuals=1,973; m=1,072,820 SNPs) or Human1M-Duo (n individuals=374; m=1,199,187 SNPs) BeadChip platforms | Non-Hispanic white | 3 population-based registries | 2,190 | 1,191 | NA |
| Jiao S, 2015 | 7,600 genes | Questionnaires and/or interviews | The Illumina HumanExome Beadchip | European descent | 7 nested case-control and 5 case-control studies in the GECCO consortium | 20,637 | 10,446 | NA |
| **Hormone replacement therapy use** | | | | | | | | |
| **Oral contraceptive use** | | | | | | | | |
| Figueiredo JC, 2011 | 770,098 SNPs | Mailed questionnaires or a telephone-based or face-to-face interview | The Illumina Human1M (n individuals=1,973; m=1,072,820 SNPs) or Human1M-Duo (n individuals=374; m=1,199,187 SNPs) BeadChip platforms | Non-Hispanic white, females only | 3 population-based registries | 2,190 | 1,191 | NA |
| **Menopausal hormone therapy use** | | | | | | | | |
| Figueiredo JC, 2011 | 770,098 SNPs | Mailed questionnaires or a telephone-based or face-to-face interview | The Illumina Human1M (n individuals=1,973; m=1,072,820 SNPs) or Human1M-Duo (n individuals=374; m=1,199,187 SNPs) BeadChip platforms | Non-Hispanic white, females only | 3 population-based registries | 2,190 | 1,191 | NA |
| Jiao S, 2015 | 7,600 genes | Questionnaires and/or interviews | The Illumina HumanExome Beadchip | European descent | 7 nested case-control and 5 case-control studies in the GECCO consortium | 20,637 | 10,446 | NA |
| Garcia-Albeniz X, 2016 | Approximately 2.7 million SNPs | Basic environmental exposures: in-person interviews and/or structured questionnaires; postmenopausal status: study-derived menopausal status, self-reported menopausal status or age >55 | HapMap II | Postmenopausal women | 10 | 10,835 | 5,419 | Fixed |

Abbreviations: BMI, body mass index; DALS, Diet, Activity and Lifestyle; FFQ, food-frequency questionnaire; G × E, gene-environment; GECCO, Genetics and Epidemiology of Colorectal Cancer Consortium; NSAID, nonsteroidal anti-inflammatory drug; SNP, single-nucleotide polymorphism.

**Supplementary table 7. SNPs With the Smallest *P* for G × E Interactions in Relation to CRC Risk That Were Reported From Each Genome-wide G × E Interaction Analysis^a^.**

| **Environmental exposure** | **Genetic variant** | **Gene (or near gene)** | **Number of studies in meta-analysis** | **Number of participants** | **Number of cases** | **Meta-analysis model** | ***P* value for interaction** | ***P* value for heterogeneity** |
| --- | --- | --- | --- | --- | --- | --- | --- | --- |
| Processed meat | | | | | | | | |
| Figueiredo JC, 2014 | rs4143094^b^ | *GATA3* | 10 | 18,404 | 9,287 | Fixed | 8.73×10^-9^ | *P=*0.78 |
| Figueiredo JC, 2014 | rs485411^b^ | *GATA3* | 10 | 18,404 | 9,287 | Fixed | 1.72×10^-8^ | *P=*0.70 |
| Figueiredo JC, 2014 | rs1269486 | *GATA3* | 10 | 18,404 | 9,287 | Fixed | 7.53×10^-8^ | *P=*0.65 |
| Total calcium intake (dietary + supplemental) | | | | | | | | |
| Du M, 2014 | rs1933755 | 6q23.1/*TMEM200A/EPB41L2* | 13 | 18,509 | 9,006 | Fixed | 1.5×10^-6^ | *P=*0.41 |
| Dietary calcium intake | | | | | | | | |
| Du M, 2014 | rs6855885 | 4q22.1/*FAM190A* | 13 | 18,509 | 9,006 | Fixed | 1.9×10^-6^ | *P=*0.55 |
| Supplementary calcium intake (single + multivitamins + antacids) | | | | | | | | |
| Du M, 2014 | rs1028166 | 4q34.3/*AGA/TENM3* | 13 | 18,509 | 9,006 | Fixed | 7.3×10^-7^ | *P=*0.29 |
| Moderate alcohol consumption (1-28 g per day) | | | | | | | | |
| Gong J, 2016 | rs9409565^c^ | 9q22.32/*HIATL1* | 14 | 16,823 | 8,058 | Fixed | 1.76×10^-8^ (permuted *P=*3.51×10^-8^) | *P=*0.96 |
| Smoking | | | | | | | | |
| Figueiredo JC, 2011 | rs2486540 | 1q44 | 3 population-based registries | 2,190 | 1,191 | NA | 3.1×10^-7^ | NA |
| Figueiredo JC, 2011 | rs2486538 | 1q44 | 3 population-based registries | 2,190 | 1,191 | NA | 3.7×10^-7^ | NA |
| Figueiredo JC, 2011 | rs538835 | 16p13.3 | 3 population-based registries | 2,190 | 1,191 | NA | 5.3×10^-7^ | NA |
| Overweight | | | | | | | | |
| Siegert S, 2013 | rs1944511 | 11q23.3 | From the PopGen biobank | 314 in stage I and 1,261 in stage II | 314 in stage I and 259 in stage II | NA | Stage I (case-only screening): *P=*3.1×10^-5^; stage II (case-control validation): unadjusted *P=*0.005; sex- and age-adjusted *P=*0.003, and multiplicity-corrected *P=*0.042 | NA |
| Aspirin use | | | | | | | | |
| Nan H, 2015 | rs2965667 | 12p12.3 | 10 | 17,187 | 8,634 | NA | 8.0×10^-7^ | *P=*0.35 |
| Aspirin and/or NSAID use | | | | | | | | |
| Nan H, 2015 | rs2965667^d^ | 12p12.3/*PIK3C2G* | 10 | 17,187 | 8,634 | NA | 4.6×10^-9^ | NA |
| Nan H, 2015 | rs10505806^d^ | 12p12.3 | 10 | 17,187 | 8,634 | NA | 5.5×10^-8^ | NA |
| Nan H, 2015 | rs16973225 | 15q25.2/*interleukin 16* | 10 | 17,187 | 8,634 | NA | 8.2×10^-9^ | NA |
| Jiao S, 2015 | 8 variants | 10p12.1/*PTCHD3* | 12 | 20,637 | 10,446 | NA | SBERIA: 0.967;  eSBERIA: 2.13×10^-7^;  GESAT: 0.00140;  coSBERIA: 0.00686;  coSKAT-O: 0.00798 | NA |
| Jiao S, 2015 | 4 variants | 17p13.2/*MINK1* | 12 | 20,637 | 10,446 | NA | SBERIA: 0.00722;  eSBERIA: 0.00201;  GESAT: 0.00460;  coSBERIA: 2.41E×10^-5^;  coSKAT-O: 5.65×10^-6^ | NA |
| Hormone replacement therapy use | | | | | | | | |
| Oral contraceptive use | | | | | | | | |
| Figueiredo JC, 2011 | rs17329226 | 3q | 3 population-based registries | 2,190 | 1,191 | NA | 7.0×10^-7^ | NA |
| Any menopausal hormone therapy | | | | | | | | |
| Garcia-Albeniz X, 2016 | rs964293 | *CYP24A1* | 10 | 10,835 | 5,419 | Fixed | Empirical Bayes: 0.11; logistic regression: 0.81; Cocktail^e^: 0.81 | Empirical Bayes: *P=*0.11; logistic regression: *P=*0.021^;^ Cocktail^e^: *P=*0.021 |
| Oestrogen plus progestogen therapy | | | | | | | | |
| Garcia-Albeniz X, 2016 | rs964293 | *CYP24A1* | 10 | 10,835 | 5,419 | Fixed | Empirical Bayes: 4.8×10^-9^; logistic regression: 1.2×10^-5^; Cocktail^e^: 1.2×10^-5^ | Empirical Bayes: *P=*0.043; logistic regression: *P=*0.028; Cocktail^e^: *P=*0.028 |
| Oestrogen-only therapy | | | | | | | | |
| Garcia-Albeniz X, 2016 | rs964293 | *CYP24A1* | 10 | 10,835 | 5,419 | Fixed | Empirical Bayes: 0.90; logistic regression: 0.38; Cocktail^e^: 0.38 | Empirical Bayes: *P=*0.27; logistic regression: *P=*0.068; Cocktail^e^: *P=*0.068 |

Abbreviations: coSBERIA, a case-only extension for eSBERIA; coSKAT-O, a case-only version of SNP-set Kernal Association Test; CRC, colorectal cancer; eSBERIA, an enhanced set-based G × E testing; G × E, gene-environment; GESAT, a G × E set association test; LD, linkage disequilibrium; NA, not available; NSAID, nonsteroidal anti-inflammatory drug; SBERIA, a Set Based gene EnviRonment InterAction test; SNP, single-nucleotide polymorphisms.

^a^ A summary of all the genome-wide G × E interaction analyses is presented in Supplementary table 6.

^b^ Genetic variants rs4143094 and rs485411 are in LD (LD r^2^ >0.6).

^c^ All the other 10 genome-wide significant SNPs were in strong LD with rs9409565 (LD r^2^ >0.8, see Supplementary table 3 of Gong J et al.)*.*

^d^ Genetic variants rs2965667 and rs10505806 are in LD, and have D’ = 1.0 and r^2^ = 0.74 in HapMap.

^e^ Cocktail method is a 2-step approach consisting a screening step to prioritize SNPs and a testing step for G × E interaction.

**Supplementary table 8. General Characteristics and Main Findings of the Systematic Reviews of Observational Studies. Suggestive Associations for G × E Interactions in Relation to CRC Risk That Were Identified by the Authors of the Original Systematic Reviews are Shown in Bold.**

| **Environmental exposure** | **Gene (genetic variant）** | **Study design** | **Comparison** | **Number of studies in systematic reviews** | **Author’s interpretation of systematic reviews** |
| --- | --- | --- | --- | --- | --- |
| **Meat** | | | | | |
| Andersen V, 2013 | ATP-binding cassette (ABC) transporters: *ABCB1, ABCC2 and ABCG2* | Prospective, population-based | Interactions between intakes of 25 g of meat per day and genotypes | 2 | A significant interaction between genetic variation in ABCB1, but not ABCC2 or ABCG2, and meat intake in relation to CRC was found. Direct transport of meat carcinogens by ABCB1 does not seem to contribute to CRC. |
| Andersen V, 2013 | Heme oxygenase-1 (*HMOX1)* A-413T | Prospective, population-based | Interactions between intakes of 25 g of red and processed meat per day and genotypes | 1 | No interaction between meat intake and HMOX1 A-413T was found, suggesting that heme from meat is not important in CRC development |
| Andersen V, 2013 | *NFKB1* -94 ins/del ATTG (rs28362491) | Prospective, population-based | Interactions between intakes of 25 g of meat per day and genotypes | 1 | Individuals with a low anti-inflammatory response increased their risk of developing CRC by meat intake in contrast to individuals with the homozygous ins-allele whose risk was unchanged by meat intake. |
| Andersen V, 2013 | O6-Methyl-guanine-DNA methyltransferase (*MGTM)* Ile143Val | Prospective, population-based | ≥ 56 vs < 56 g/day of meat intake in analyses stratified by genotypes | 1 | Individuals with low DNA repair capacity of oxidative DNA damage are at a high risk of CRC by meat intake in contrast to individuals with the homozygous wildtype, whose risk seems to be unchanged by meat intake. |
| Andersen V, 2015 | Cooking carcinogens and mutagens: *NAT1, NAT2* | Prospective | High vs low servings of meat per day in analyses stratified by genotypes | 5 | One small study found interaction between the number of servings per day and NAT2 acetylator status, no association was found between the amount of total or processed meat intake or number of servings and NAT1 or NAT2 status in relation to the risk of CRC in 3 other studies. |
| Andersen V, 2015 | Arachidonic acid pathway: *PTGS2* G-765C (rs20417) | Prospective | Interactions between intakes of 25 g of red and processed meat per day and genotypes | 2 | Individuals carrying the G-765C C-variant allele were at 8 % increased risk of CRC per 25 g red and processed meat per day in contrast to the homozygous wild-type carriers whose risk of CRC was unaffected by meat intake |
| Andersen V, 2015 | Transport proteins: *ABCB1, ABCC2* and *ABCG2* | Prospective | Interactions between intakes of 25 g of red and processed meat/day and genotypes | 3 | Interactions between meat intake and polymorphisms in ABCB1 in relation to the risk of CRC were found in a prospective cohort, whereas no interactions were found for the 2 other transport proteins, ABCC2 and ABCG2. |
| Andersen V, 2015 | Cytokines: *IL10 rs3024505*, *IL10* C-592A and 3 functional *IL1B* polymorphisms (C-3737T, G-1464C and T-31C) | Prospective | Interactions between intakes of 25 g of red and processed meat/day and genotypes | 2 | Interaction between meat intake and the marker polymorphism near IL10 rs3024505 was found in a prospective cohort, whereas no interaction was found with the functional IL10 C-592A nor with 3 functional IL1B polymorphisms. |
| Andersen V, 2015 | Transcription factors: *NR1I2, NR1H2 and NFKB1* -94 ins/del (rs2836249) | Prospective | Interactions between intakes of 25 g of red and processed meat/day and genotypes | 1 | No interactions were found between meat intake and the genes NR1I2 and NR1H2 encoding PXR and LXR in relation to CRC.  Interactions were found between meat intake and NFKB1 (encoding the anti-inflammatory subunit p50/p105 of NFκB) −94 ins/del (rs2836249) in relation to CRC risk. |
| Andersen V, 2015 | Heme oxygenase: *HMOX1* A-413T rs2071746 | Prospective | Interactions between intakes of 25 g of red and processed meat/day and genotypes | 1 | No interactions were found between the functional HMOX1 A-413T (rs2071746) polymorphism and meat intake in relation to CRC. |
| Andersen V, 2015 | DNA repair*: MSH3* T1036A (rs26279) *and R940Q* (rs184967) | Prospective | Variant carriers vs homozygous wildtype carriers in analyses stratified by processed meat intakes (≥10.1 vs <10.1 g/day) | 1 | Interpretation of the interaction result was not possible because possible functional effects of the polymorphisms were not known. |
| Andersen V, 2015 | DNA repair*: XPC Lys939Gln* | Prospective or case-control | Interactions between intakes of 100 g of red meat per day and genotypes;  high vs low well-done red meat intake in analyses stratified by genotypes | 2 | Interaction between the intake of red meat and XPC Lys939Gln and a suggestive interaction between the intake of processed meat and XPA A23G was found. |
| Shin A, 2010 | *NAT1 and NAT2* | Prospective or case-control | See Table 1 of Shin A, et al., 2010 | 10 | Several studies have reported that increased CRC risk was observed with high intakes of red meat, fried meat, processed meat, meat and fat dietary pattern, or high meat-mutagen index among subjects with rapid NAT1 or NAT2 acetylator genotypes. In contrast, some other studies did not report any apparent effect of interaction between NAT1 or NAT2 genotypes and meat intake on CRC risk. |
| Shin A, 2010 | *Cytochrome P450: CYP1A2, CYP2E1, CYP1B1, CYP2C9* | Prospective or case-control | High vs low meat intake in analyses stratified by genotypes | 7 | The associations were not consistent across studies. |
| Shin A, 2010 | *GSTM1, GSTP1 and GSTT1* | Prospective | High vs lowest meat intake in analyses stratified by genotypes;  high vs low white meat-mutagen index in analyses stratified by genotypes | 5 | The associations were not consistent across studies. |
| Shin A, 2010 | *Sulfotransferase (SULT)* | Prospective | High vs low frequency of meat intake in analyses stratified by genotypes | 2 | Two studies found a suggestive effect modification of the association between meat intake and CRC risk by the polymorphisms. |
| **Fish** | | | | | |
| Andersen V, 2013 | 23 polymorphisms in the arachidonic acid pathway | Prospective, population-based | More than once per week vs less than once per week of fish intake in analyses stratified by genotypes | 1 | No significant interactions were found. |
| Lenihan-Geels G, 2016 | *COX1* (Phe17Leu) | Case-control | Reference: PP and <1 time per week of fish intake | 1 | Low EPA/DHA intake associated with higher colon cancer risk in variant allele carriers only. |
| Lenihan-Geels G, 2016 | *PGES* (rs7873087) | Case-control | Reference: AA and <1 time per week of fish intake | 1 | Carriers of T allele have lower CRC risk with higher fish intake. |
| Lenihan-Geels G, 2016 | *EP4* (Val294Ile) | Case-control | Reference: Val/Val and <1 time per week of fish intake | 1 | Carriers of Ile variant showed correlation between high fish intake and higher CRC risk. |
| **Cruciferous vegetables (isothiocyanates)** | | | | | |
| Andersen V, 2013 | GST activity: *GSTM1 and GSTT1* | Prospective, population-based | High vs low intake of isothiocyanates in analyses stratified by GST genotypes | 1 | Isothiocyanates from cruciferous vegetables protect against CRC in individuals with low GST activity. |
| **Milk and dairy products** | | | | | |
| Kostner K, 2009 | *VDR* Bsm1 | Case-control | Reference: homozygous wildtype genotype and low low-fat dairy products (servings/day) | 1 | Positive effects on cancer risk were reported for patients with high levels of consumption of low-fat dairy products (OR=0.61). With regard to colon cancer, high levels of intakes of low-fat dairy products showed a protective effect for the BB genotype. |
| Kostner K, 2009 | *VDR* Poly (A) | Population-based case-control | Reference: homozygous wildtype genotype and low low-fat dairy products (servings/day) | 1 | For colon cancer, high levels of intake of low-fat dairy products reduced risk of cancer for the SS genotype, although the *P* for interaction was not statistically significant. |
| **Cereal** | | | | | |
| Andersen V, 2013 | *IL10 and ABCC2* | Prospective, population-based | Per 50 g/day of cereal intake in analyses stratified by genotypes | 2 | No significant associations between milk, cereal or dairy and IL10 and ABCC2 gene polymorphisms. |
| **Fiber** | | | | | |
| Andersen V, 2013 | *IL10* C-592A | Prospective, population-based | Reference: carriers of the homozygous wildtype and eating <17.0 g of fiber per day | 1 | The increased risk caused by carrying the IL10 low-activity variant C-592-A-allele can be overcome by high fiber intake. Thus, high intake of fiber seems to protect against CRC among individuals with genetically determined low IL10 activity. |
| Kostner K, 2009 | *VDR* Fok1 | Case-control | Reference: homozygous wildtype genotype and low sucrose-to-fiber ratio | 1 | The importance of Fok1 polymorphisms for cancer risk may strongly depend on additional factors including VDR haplotype combinations, other genetic factors, and other cancer-specific risk factors. |
| **Alcohol consumption** | | | | | |
| Andersen V, 2013 | *PPARG* Pro12Ala | Prospective, population-based | Interactions between consumption of 10 g of alcohol per day and genotypes | 1 | The interaction has not been reproduced for other CRC cohorts, but the finding is indirectly supported by the fact that interaction between PPARG Pro12Ala and alcohol intake was also found in relation to breast cancer. |
| **Sharp L, 2004** | ***MTHFR C677T*** | **Prospective** | **Largest vs smallest quantity of alcohol intake in analyses stratified by genotypes; ever vs never drinking in analyses stratified by genotypes** | **3** | **In 2 studies, 677TT homozygote subjects with the highest alcohol intake had the highest cancer risk.** |
| Sharp L, 2004 | *MTHFR A1298C* | Prospective | Reference: ever vs never alcohol intake in analyses stratified by genotypes | 1 | No interactions were observed between A1298C and “ever” or “never” consuming alcohol. |
| **Moderate alcohol consumption (<30 g per day)** | | | | | |
| Klarich DS, 2015 | *ADH and ALDH* | Case-control | Highest vs lowest alcohol intake in analyses stratified by genotypes | 3 | Some significant results suggest that the development of CRC is dependent on the interaction of gene and environment. Genetic susceptibility may contribute additional CRC risk for those consuming alcohol. |
| Klarich DS, 2015 | DNA repair*: CYP2E1* and *XRCC1* | Case-control | ≥ 2.0 units or < 2.0 units of alcohol consumption per day vs nondrinkers in analyses stratified by genotypes;  >20 g/d or ≤20 g/d of alcohol consumption in analyses stratified by genotypes | 3 | Some significant results suggest that the development of CRC is dependent on the interaction of gene and environment. Genetic susceptibility may contribute additional CRC risk for those consuming alcohol. |
| **Folate intake or plasma folate level** | | | | | |
| Andersen V, 2013 | *MTHFR* A1298C | Case-control | Low-risk (low folate and methionine intake and high alcohol use) vs high-risk dietary pattern in analyses stratified by genotypes | 1 | The risk associated with carrying the MTHFR A1298C variant allele can be overcome by a high intake of folate and methionine and low alcohol intake. |
| Sharp L, 2004 | *MTHFR A1298C* | Prospective | Reference: AA + CC and ≥400μg/day of folate intake | 1 | White 1298CC subjects who consumed less than 400 ng of folate per day had a greater reduced cancer risk than those whose folate intake was higher. |
| **Sharp L, 2004** | ***MTHFR C677T*** | **Prospective** | **Reference: ala/ala and ala/val with adequate folate level;**  **CC with the lowest tertile of food or total folate;**  **reference: val/ala and ala/ala with low folate or methionine;**  **reference: CC+TT with ≥400 μg/day of folate intake** | **5** | **In 4 of 5 genotype-diet interaction studies, 677TT subjects who had higher folate levels (or a "high-methyl diet") had the lowest cancer risk.** |
| Sharp L, 2004 | *MTR* | Prospective | Reference: MRT asp/asp or gly/asp with tertile 2 and 3 of folate levels (ng/ml) | 1 | Replication is needed. The roles of folate-pathway genes, folate, and related dietary factors in colorectal neoplasia are complex. |
| Eicbbolzer M, 2001 | *MTHFR* and MTR | Prospective | Homozygous mutation vs homozygous normal or heterozygous genotypes in analyses stratified by plasma folate levels [deficient plasma folate level (<3 ng/ml) vs adequate folate level];  high vs low folate intake in analyses stratified by genotypes | 4 | The association between folate intake and colorectal neoplasia seems to be modulated by dietary factors such as alcohol, and methionine. Furthermore, MTHFR polymorphisms and adequate folate intake a decreased risk of CRC has been observed. However, no inverse association was observed in those with a diet inadequate in folate, high in alcohol, or low in methionine. |
| **Vitamin D intake** | | | | | |
| Kostner K, 2009 | *VDR* Bsm1 | Hospital-based case-control | Reference: homozygous wildtype genotype and highest vitamin D intake (IU) | 2 | Vitamin D intake seem to influence the CRC risk. But the results are controversial. Therefore, when analysing the VDR genotype, differences in gender should be taken into consideration. |
| Kostner K, 2009 | *VDR* Poly (A) | Population-based case-control | Reference: homozygous wildtype genotype and low vitamin D intake | 1 | For colon cancer, high levels of dietary intake of vitamin D reduced risk of cancer for the SS genotype, although the *P* for interaction was not statistically significant. |
| Kostner K, 2009 | *VDR* Cdx2 | Case-control | NA | 1 | Cdx2 polymorphism was not independently associated with either colon or rectal cancer, nor did it modify associations of dietary vitamin D with colon or rectal cancer. |
| **Riboflavin intake** | | | | | |
| Sharp L, 2004 | *MTHFR* | Cross-sectional | Lowest vs highest riboflavin quartile in analyses stratified by genotypes | 1 | The lowest relative risk for cancer among TT persons with the highest riboflavin intake was found. Genotype-folateriboflavin combinations were not considered. |
| **Vitamin C intake** | | | | | |
| Andersen V, 2013 | *MGTM Ile143Val* | Prospective, population-based | Reference: homozygous wildtype carriers and low intake | 1 | Individuals with genetically determined low DNA repair capacity are at a high risk of CRC by low intake of vitamin C, vitamin E and carotene. |
| **Vitamin E intake** | | | | | |
| Andersen V, 2013 | *MGTM Ile143Val* | Prospective, population-based | Reference: homozygous wildtype carriers and low intake | 1 | Individuals with genetically determined low DNA repair capacity are at a high risk of CRC by low intake of vitamin C, vitamin E and carotene. |
| **Carotene intake** | | | | | |
| Andersen V, 2013 | *MGTM Ile143Val* | Prospective, population-based | Reference: homozygous wildtype carriers and low intake | 1 | Individuals with genetically determined low DNA repair capacity are at a high risk of CRC by low intake of vitamin C, vitamin E and carotene. |
| **Calcium intake** | | | | | |
| Andersen V, 2013 | *VDR* Fok1*, Bsm1 and poly A* | Prospective, population-based | Reference: homozygous wildtype genotype and low calcium intake;  homozygous wildtype genotype and high calcium intake | 2 | A high intake of calcium can overcome the risk conferred by a deficient VDR. |
| Kostner K, 2009 | *VDR* Fok1 | Population-based case-control | Reference: homozygous wildtype genotype and low calcium intake;  homozygous wildtype genotype and high calcium intake | 1 | The importance of Fok1 polymorphisms for cancer risk may strongly depend on additional factors including VDR haplotype combinations, other genetic factors, and other cancer-specific risk factors. |
| Kostner K, 2009 | *VDR Bsm*1 | Hospital-based case-control | Reference: homozygous wildtype genotype and highest tertile of calcium intake (mg) | 1 | Vitamin D intake seem to influence the CRC risk. But the results are controversial. Therefore, when analysing the VDR genotype, differences in gender should be taken into consideration. |
| Kostner K, 2009 | *VDR* Poly (A) | Population-based case-control | Reference: homozygous wildtype genotype and low calcium intake | 1 | The SS genotype has been shown to be associated with a significant 40% risk reduction of rectal cancer when calcium intake was low (p interaction=0.01 for calcium interaction). |
| Kostner K, 2009 | *VDR* Cdx2 | Case-control | NA | 1 | Cdx2 polymorphism was not independently associated with either colon or rectal cancer, nor did it modify associations of dietary calcium with colon or rectal cancer. |
| **Fat, saturated fatty acids, monounsaturated fatty acids, n-6 PUFA, n-3 PUFA, LCn-3 PUFA and cholesterol intake** | | | | | |
| Andersen V, 2013 | *PPARG* Pro12Ala and C161T | Case-control | Highest vs lowest intakes of fat, saturated fatty acids, monounsaturated fatty acids, n-6 PUFA, n-3 PUFA, or cholesterol in analyses stratified by genotypes | 1 | No significant interactions between PPARG Pro12Ala and C161T polymorphisms and intake of fat, saturated fatty acids, monounsaturated fatty acids, n-6 PUFA, n-3 PUFA, or cholesterol in relation to CRC risk. |
| Kostner K, 2009 | *VDR* Fok1 | Prospective | Reference: homozygous wildtype genotype and low dietary fat intake (<41.54 g/day) | 1 | The importance of Fok1 polymorphisms for cancer risk may strongly depend on additional factors including VDR haplotype combinations, other genetic factors, and other cancer-specific risk factors. |
| Kostner K, 2009 | *VDR* Cdx2 | Case-control | NA | 1 | Cdx2 polymorphism was not independently associated with either colon or rectal cancer, nor did it modify associations of dietary fat with colon or rectal cancer. |
| Corella D, 2012 | *XRCC1-194, XRCC1-399 and XRCC3-241* | Case-control | Reference: low n-6/n-3 ratio with XRCC1 Arg/Arg Arg/Arg or Arg/Gln | 1 | The effect of the XRCC1 polymorphisms on colo-rectal cancer risk is modulated by the n-6/n-3 ratio. A high n-6/n3 ratio increased the riks in subjects carrying the XRCC1 Trp 194 allele or the Arg 399 allele. |
| Corella D, 2012 | *XRCC1 rs1799782 and*  *rs25487; PARP rs1136410 and rs3219145; OGG1 rs1052133; XPD rs1799793 and rs13181* | Nested case-control | Reference: low marine n-3 PUFA intake with *PARP Val/Val* genotype | 1 | The PARP Val762Ala SNP modified the association between marine n-3 PUFA and rectal cancer risk, with no evidence of interaction among colon cancer. Carriers of the 762Ala allele have higher rectal. |
| Lenihan-Geels G, 2016 | *ALOX12* (rs11571339) | Case-control | Reference: AA and low n-3 PUFA intake | 1 | Certain individuals may benefit largely from including LCn-3 PUFA in their diets while others do not, as demonstrated by polymorphisms in ALOX12, ALOX15  and PGES genes. |
| Lenihan-Geels G, 2016 | *ALOX15* (rs11568131) | Case-control | Reference: GG and low n-3 PUFA intake | 1 | Certain individuals may benefit largely from including LCn-3 PUFA in their diets while others do not, as demonstrated by polymorphisms in ALOX12, ALOX15  and PGES genes. |
| Lenihan-Geels G, 2016 | *COX1* (rs10306110) | Case-control | Reference: homozygous wildtype genotype and low EPA/DHA intake in analyses stratified by genotypes | 1 | Certain individuals may benefit largely from including LCn-3 PUFA in their diets while others do not, as demonstrated by polymorphisms in ALOX12, ALOX15  and PGES genes. |
| **Coffee and tea intake** | | | | | |
| Cornelis MC, 2014 | *GSTM1* (Del) | Prospective | Reference: None intake of coffee with the null genotype | 1 | No overall protective effect of coffee or effect modification by genetic variation in  GSTM1, NAT2, or CYP1A2 was found. |
| Cornelis MC, 2014 | *CYP1A2* (rs762551) | Prospective | Reference: non/low coffee and tea intake with a fast *CYP1A2* activity | 1 | No overall protective effect of coffee or effect modification by genetic variation in  GSTM1, NAT2, or CYP1A2 was found. |
| Cornelis MC, 2014 | *NAT2* (rs1801279, rs1799930, rs1799931, rs1799929) | Prospective | Reference: non/low consumers with fast *NAT2* acetylation status | 1 | No overall protective effect of coffee or effect modification by genetic variation in  GSTM1, NAT2, or CYP1A2 was found. |
| **Aspirin use** | | | | | |
| Andersen V, 2014^a^ | Wnt/β-catenin signaling: *MYC* rs6983267 | Prospective cohort | Users vs non-users in analyses stratified by genotypes | 1 | The protective effect of aspirin was confined to homozygous carriers of the protective T allele of rs6983267 who constitute ca. 25% of the population. |
| **NSAID use** | | | | | |
| Andersen V, 2014^a^ | Arachidonic acid pathway: *PTGS1* | Case-control | Users vs non-users in analyses stratified by genotypes | 1 | Interactions between NSAID use and PTGS1 rs3842787, rs10306135 and rs6478565 in relation to rectal cancer were found. |
| Andersen V, 2014^a^ | Arachidonic acid pathway: *PTGS2* | Prospective cohort or case-control | Users vs non-users in analyses stratified by genotypes | 5 | No interaction between use of NSAIDs and PTGS-2 in relation to CRC risk was detected. |
| Andersen V, 2014^a^ | Arachidonic acid pathway: *ALOX15* | Case-control | Users vs non-users in analyses stratified by genotypes | 1 | Interactions with NSAID use were found for ALOX15 rs2619112, ALOX5AP rs9508832, rs9315053, rs4075692 and rs9551960 in relation to rectal cancer and ALOX5AP rs17239025 in relation to colon cancer. |
| Andersen V, 2014^a^ | Arachidonic acid pathway: *PPARD* and *PPARG* | Prospective cohort or case-control | Users vs non-users in analyses stratified by genotypes | 3 | No interactions between NSAIDs and PPARD or PPARG were found. |
| Andersen V, 2014^a^ | Arachidonic acid pathway: *PLA2G1B* rs2070873 | Case-control | Users vs non-users in analyses stratified by genotypes | 1 | Interaction between NSAID use and PLA2G1B rs2070873 was found in relation to CRC. The homozygous wildtype carriers benefitted more from NSAID use than variant allele carriers. |
| Andersen V, 2014^a^ | Arachidonic acid pathway: *ABCB1, ABCC2* and *SLC22A4* | Prospective cohort | Users vs non-users in analyses stratified by genotypes | 3 | Interactions between NSAID use and polymorphisms in ABCB1, ABCC2 and SLC22A4 in relation to risk of CRC were found in a prospective cohort. |
| **Andersen V, 2014^a^** | **Cytokines: *IL10* rs3024505 and rs1800872** | **Prospective cohort or case-control** | **Users vs non-users in analyses stratified by genotypes** | **2** | **Two different studies found interaction between NSAID use and genetic variation in or close to IL10.** |
| Andersen V, 2014^a^ | Cytokines: IL1B, LEP and IL6 | Prospective cohort or case-control | Users vs non-users in analyses stratified by genotypes | 3 | NSAID use was more beneficial for those who had the highest risk of CRC. Interactions between NSAID use and IL1B, LEP and IL6 in relation to CRC were suggested. |
| Andersen V, 2014^a^ | *TCF7L2* rs7903146 | Case-control | Users vs non-users in analyses stratified by genotypes | 1 | The TCF7L2 rs7903146 T-allele carriers were at reduced risk of CRC by NSAID use in contrast to the homozygous wildtype C-allele carriers (reference). |
| Andersen V, 2014^a^ | *SMAD7* | Case-control | Users vs non-users in analyses stratified by genotypes | 1 | No interaction between NSAID use and SMAD7 in relation to CRC was found. |
| Andersen V, 2014^a^ | MAP3K10 rs892117, and MAPK1 rs2548663, MAPK1 rs2298432, MAPK12 rs2272857 and MAPK14 rs851016 | Case-control | Users vs non-users in analyses stratified by genotypes | 1 | Interactions between NSAID use and MAP3K10 rs892117 in relation to risk of colon cancer, and between NSAID use and MAPK1 rs2548663, MAPK1 rs2298432, MAPK12 rs2272857 and MAPK14 rs851016 in relation to rectal cancer were found. |
| Andersen V, 2014^a^ | *NFKB1 rs230490, IKBKB rs6474387 and rs11986055* | Prospective cohort or case-control | Users vs non-users in analyses stratified by genotypes | 2 | Interactions were found between NSAID use and NFKB1 rs230490 in relation to risk of rectal cancer, and NSAID use and IKBKB rs6474387 and rs11986055 in relation to risk of colon cancer. |
| Andersen V, 2014^a^ | *TYK2 rs280521, TYK2 rs280521, and STAT6 rs324011* | Case-control | Users vs non-users in analyses stratified by genotypes | 1 | Interactions between NSAID and the marker polymorphisms TYK2 rs280521 in relation to colon cancer, and NSAID use and TYK2 rs280521, STAT6 rs324011 and JAK2 rs10815160 in relation to rectal cancer were found. |
| Andersen V, 2014^a^ | *NR1I2 and NR1H2* | Prospective cohort | Users vs non-users in analyses stratified by genotypes | 1 | No interactions were found between NSAIDs and NR1I2 or NR1H2, encoding the xenobiotic receptors, members of the nuclear receptor superfamily, PXR and LXR, in relation to CRC in a prospective cohort. |
| Andersen V, 2014^a^ | *CYP2C9, CYP2C8* and *UGT1A6* | Case-control | Users vs non-users in analyses stratified by genotypes | 2 | Enhanced protection by use of the NSAID ibuprofen among slower metabolising CYP2C9 variant carriers compared to fast metabolisers was found. Other studies found no interaction between NSAID use and polymorphisms in UGT1A6, CYP2C8 and CYP2C9 in relation to risk of CRC. |
| Andersen V, 2014^a^ | *Toll like receptors (TLR): TLR2 rs7656411 and TLR3 rs11721827* | Case-control | Users vs non-users in analyses stratified by genotypes | 1 | One case–control study found that carriers of the variant alleles of TLR2 rs7656411 and TLR3 rs11721827 benefitted more from NSAID use than the homozygous wildtype carriers. |
| Andersen V, 2014^a^ | *TERT-CLPTM1L rs2853668* | Case-control | Users vs non-users in analyses stratified by genotypes | 1 | TERT-CLPTM1L rs2853668 homozygous variant allele carriers benefitted more from NSAID use than wildtype carriers. Interaction between NSAID use and TERT-CLPTM1L in relation to risk of CRC was suggested. |
| Andersen V, 2014^a^ | *DLG5* G113A | Prospective cohort | Users vs non-users in analyses stratified by genotypes | 1 | The DLG5 G113A A-allele carriers benefitted more from NSAID use than homozygous wildtype carriers. Interaction between NSAID use and DLG5 in relation to risk of CRC was suggested. |
| Andersen V, 2014^a^ | *TP53* Arg72Pro and *p53PIN3* | Case-control | Users vs non-users in analyses stratified by genotypes | 1 | For both polymorphisms, it was found that the homozygous wildtype carriers benefitted more from use of NSAIDs than variant allele carriers. |
| Cross JT, 2007 | *COX2 (G10335A, C1629G, G3050C, A401G, 5209T>G, T8473C, G926C, A9850G, Val511Ala)* | Case-control | Users vs non-users in analyses stratified by genotypes | 3 | No statistically significant interactions have been reported for polymorphisms in COX2; however, an interaction with COX2 -765G>C approached significance (*P=*0.07) in 1 study. |
| Cross JT, 2007 | *UGT1A6* (Thr181Ala + Arg184Ser) | Case-control | Users vs non-users in analyses stratified by genotypes | 1 | Statistically significant interactions were reported for UGT1A6 (Thr181Ala+Arg184Ser or Arg184Ser alone). |
| Cross JT, 2007 | *CYP2C9* | Case-control | Users vs non-users in analyses stratified by genotypes | 1 | Risk reduction with ASA use stronger among those with variant allele; no interaction with ibuprofen use. |
| **BMI, physical activity, energy intake, energy expenditure** | | | | | |
| Kostner K, 2009 | *VDR* Fok1 | Prospective | Reference: homozygous wildtype genotype with low BMI or low physical activity | 1 | The importance of Fok1 polymorphisms for cancer risk may strongly depend on additional factors including VDR haplotype combinations, other genetic factors, and other cancer-specific risk factors. |
| Kostner K, 2009 | *VDR* Poly (A) | Population-based case-control | Reference: homozygous wildtype genotype with low BMI or physical activity | 1 | Obese individuals with the SS genotype had a greater risk of colon cancer (OR=3.5), though the association between energy intake and colon cancer appears to be driven more by energy intake than by the poly (A) VDR genotype. |
| **Smoking** | | | | | |
| Karahalil B, 2012 | XRCC1 | Case-control | Smokers vs nonsmokers in analyses stratified by genotypes | 4 | The XRCC1 Arg399Gln gene polymorphism was not found to be interacting significantly with tobacco consumption on cancer susceptibility (interaction *P >*0.05, respectively). |
| Houlston RS, 2001 | *GSTM1 and GSTT1* | Prospective | Smokers vs nonsmokers in analyses stratified by genotypes | 3 | The results of the studies are not consistent. |
| Simonds NI, 2016 | *NAT2* | Prospective | Ever (current/former) smokers vs never smoked, >27 years smoked vs never smoked, >22 pack-years smoked vs never smoked in analyses stratified by genotypes | 1 | NA |
| **Selenium intake or blood selenium level** | | | | | |
| Andersen V, 2013 | Genes encoding selenoproteins and genetic polymorphisms in selenoproteins | Prospective or case-control | NA | 2 | Genetic variations in genes encoding selenoproteins were not associated with CRC risk and blood selenium level, and no interaction was found between selenium intake and genetic polymorphisms in selenoproteins in relation to CRC risk. |
| **Sunshine exposure** | | | | | |
| Kostner K, 2009 | *VDR* Bsm1 | Case-control | BB or heterozygote vs bb genotype in analyses stratified by sunshine exposure status (high vs low) | 1 | Positive effects on cancer risk were reported for patients with high levels of sunshine exposure (OR=0.62 for men diagnosed when <60 years). |

Abbreviations: BMI, body mass index; CRC, colorectal cancer; G × E, gene-environment; DHA, Docosahexaenoic acid; EPA, Eicosapentaenoic acid; LCn-3 PUFA, long chain omega-3 polyunsaturated fatty acids; NA, not available; NSAID, nonsteroidal anti-inflammatory drug; PUFA, polyunsaturated fatty acids; VDR, vitamin D receptor; vs, versus.

^a^ The component studies in the systematic reviews vary considerably in design and definition of NSAIDs use (see Table 2 in Andersen V, 2014).
